# In utero electroporation induces cell death and alters embryonic microglia morphology and expression signatures in the developing hypothalamus

**DOI:** 10.1186/s12974-018-1213-6

**Published:** 2018-06-12

**Authors:** Jessica M. Rosin, Deborah M. Kurrasch

**Affiliations:** 1 Department of Medical Genetics, Cummings School of Medicine, University of Calgary, 3330 Hospital Drive NW, Room HS2215, Calgary, Alberta T2N 4N1 Canada; 2 Alberta Children’s Hospital Research Institute, University of Calgary, Calgary, Alberta Canada; 3 Hotchkiss Brain Institute, University of Calgary, Calgary, Alberta Canada

**Keywords:** Microglia, Hypothalamus, In utero electroporation, Inflammation

## Abstract

**Background:**

Since its inception in 2001, in utero electroporation (IUE) has been widely used by the neuroscience community. IUE is a technique developed to introduce plasmid DNA into embryonic mouse brains without permanently removing the embryos from the uterus. Given that IUE labels cells that line the ventricles, including radial fibers and migrating neuroblasts, this technique is an excellent tool for studying factors that govern neural cell fate determination and migration in the developing mouse brain. Whether IUE has an effect on microglia, the immune cells of the central nervous system (CNS), has yet to be investigated.

**Methods:**

We used IUE and the *pCIG2*, *pCIC-Ascl1*, or *pRFP-C-RS* expression vectors to label radial glia lining the ventricles of the embryonic cortex and/or hypothalamus. Specifically, we conducted IUE at E14.5 and harvested the brains at E15.5 or E17.5. Immunohistochemistry, along with cytokine and chemokine analyses, were performed on embryonic brains with or without IUE exposure.

**Results:**

IUE using the *pCIG2*, *pCIC-Ascl1*, or *pRFP-C-RS* vectors alone altered microglia morphology, where the majority of microglia near the ventricles were amoeboid and displayed altered expression signatures, including the upregulation of Cd45 and downregulation of P2ry12. Moreover, IUE led to increases in P2ry12^−^ cells that were Iba1^+^/IgG^+^ double-positive in the brain parenchyma and resembled macrophages infiltrating the brain proper from the periphery. Furthermore, IUE resulted in a significant increase in cell death in the developing hypothalamus, with concomitant increases in cytokines and chemokines known to be released during pro-inflammatory states (IL-1β, IL-6, MIP-2, RANTES, MCP-1). Interestingly, the cortex was protected from elevated cell death following IUE, implying that microglia that reside in the hypothalamus might be particularly sensitive during embryonic development.

**Conclusions:**

Our results suggest that IUE might have unintended consequences of activating microglia in the embryonic brain, which could have long-term effects, particularly within the hypothalamus.

**Electronic supplementary material:**

The online version of this article (10.1186/s12974-018-1213-6) contains supplementary material, which is available to authorized users.

## Background

Microglia are the resident immune cells of the central nervous system (CNS). Murine microglia are generated from yolk sac erythro-myeloid progenitors (EMPs), which are a distinct lineage from hematopoietic stem cells [1–3]. During embryonic development, tissue-resident macrophages develop from EMPs that travel from the yolk sac to the developing CNS to generate microglia [1–3]. Changes in cellular expression signatures appear amid the transition from macrophage to microglia, including the downregulation of Cd45 and the eventual expression of the microglia-specific marker P2ry12 [4]. Following the establishment of the blood-brain barrier (BBB) embryonically, peripherally circulating myeloid cells are thought to rarely infiltrate the brain parenchyma under normal conditions [2, 5]. Microglia continue to mature across development, altering their morphology and gene expression signatures throughout embryonic, postnatal, and adult stages [3, 6], leading to speculation of differing roles for microglia across developmental stages.

Increasingly, microglia are being shown to play a role in neural development. For example, in the embryonic brain, microglia influence neural progenitor maintenance [7], as well as progenitor engulfment and elimination, which serves to signal the termination of neurogenesis [8]. During postnatal development, microglia are involved in dendritic spine formation [9] and synaptic pruning [10–12], both of which can influence neuronal connectivity. Combined, a growing body of literature shows that microglia functionally contribute to embryonic and postnatal neural development, highlighting the importance of this immune cell in normal healthy CNS development. Moreover, recent reports have demonstrated that inflammation, in the form of infection or stress, can lead to microglia activation and destructive consequences, such as learning and memory impairments and behavioral alterations [13–19].

In utero electroporation (IUE) is a technique developed to introduce plasmid DNA into embryonic mouse brains, while the animals are still alive in the uterus [20–22]. Many groups have now shown that electroporated embryos can continue to develop normally in utero and that most electroporated embryos survive to birth [20–22], thereby suggesting minimal effects on the developing fetus. Given that IUE labels cells in the ventricular zone, including radial fibers and migrating neuroblasts, this technique is an excellent tool for studying neural cell fate determination and migration in the developing mouse brain. A large body of research has used IUE to show how telencephalic structures, such as the cortex and hippocampus, develop and how the knockdown or overexpression of specific genes can influence and disrupt normal processes [22–25]. The IUE procedure also has more recently been adapted for the developmental study of diencephalic brain regions, such as the thalamus and hypothalamus [26–28]. IUE also is now employed to examine the contribution of genetics to changes in observed behavioral outputs [29]. The use of IUE in microglia studies is currently minimal, given that microglia cannot be targeted directly using this approach. At present, IUE has been used to show that microglia regulate the number of neural precursors in the developing cortex [8] and that microglia contact itself can induce synapse formation in the developing somatosensory cortex following labeling of projections using IUE [9]. The use of IUE in microglia studies has also shown that developing neural progenitors in the cortex play a role in microglia migration and localization [30]. Considering the utility of IUE as a tool to ectopically express DNA or knockdown gene expression, it is likely that this approach will continue to be employed in the microglia field.

Given our interest in radial glia and microglia interactions, and since we routinely employ IUE to label radial glial cells, we wanted to explore any potential effects of the IUE technique on microglia. Following *pCIG2*, *pCIC-Ascl1*, or *pRFP-C-RS* IUE, embryonic brains demonstrated high numbers of amoeboid microglia that displayed altered expression signatures within 24 h following electroporation, including the upregulation of Cd45 and downregulation of P2ry12. IUE also resulted in a significant increase in cell death in the developing hypothalamus, including changes in cytokines and chemokines known to be released during pro-inflammatory states. Taken together, our results demonstrate that embryonic microglia become activated following IUE, and suggest that the hypothalamus is particularly sensitive to inflammation.

## Methods

### Mouse strains

CD1 mice (Charles River) were used for all experiments. Animal protocols were approved by the University of Calgary Animal Care Committee and followed the Guidelines for the Canadian Council of Animal Care.

### In utero electroporation (IUE)

The IUE procedure has been described elsewhere [27]. In brief, the *pCIG2* expression vector, which contains a β-actin promoter/CMV enhancer and an IRES–EGFP cassette, was used for IUE shown in the primary figures. In addition, the *pCIC-Ascl1* expression vector, which contains a β-actin promoter/CMV enhancer upstream of the *Ascl1* sequence and an IRES–mCherry cassette, and the *pRFP-C-RS* expression vector (TR30014, OriGene), which contains a CMV promoter and a tRFP cassette, were used in Additional file 1: Figures S1 and S5. Females were anesthetized with 5 L/min isoflurane, which was decreased to 2.5 L/min during surgery, with oxygen flow at 1 L/min. To prevent infection and pain post-surgery, the antibiotic enrofloxacin (Baytril) and the pain killer buprenorphine were administered subcutaneously to anesthetized females. Using an Eppendorf FemtoJet 4i microinjector (VWR) and a Narishige 3-axis M152 micromanipulator (Leica), *pCIG2* DNA was injected at a concentration of 0.5–0.7 μg/μL into the lateral ventricle of E14.5 brains. Following DNA injection, 7 mm BTX platinum plated electrodes (Harvard Apparatus) and a BTX ECM 830 Electro Square Porator (Harvard Apparatus) were used to pulse (45 V, 50 ms) embryonic brains five times, separated by intervals of 950 ms. Once the embryos were placed back inside the pregnant dam, the cavity was filled with warm saline and the peritoneum was sutured shut, which was followed by suturing closed the abdominal wall. Following the stop of anesthesia, 2 mL of Ringer’s solution was injected into the back of the pregnant female, which was placed on a heating pad to aid in recovery.

### Immunohistochemistry

Twenty-four or 72 h following IUE, E15.5, or E17.5 brains were collected in ice-cold phosphate-buffered saline (PBS) and fixed in 4% paraformaldehyde (PFA) overnight at 4 °C. The brains were then washed in PBS and equilibrated in 20% sucrose/PBS overnight at 4 °C. Brains were embedded in Clear Frozen Section Compound (VWR, 95057-838) and cryosectioned (10–20-μm sections). For immunohistochemistry (IHC), cryosections were rehydrated in PBS, washed with PBT (PBS with 0.1% Triton-X), blocked using 5% normal donkey or goat serum (NDS or NGS, Sigma) for 1 h at room temperature (RT), and exposed to rabbit anti-Fezf1 (1:100, Fitzgerald 70R-7693), chicken anti-GFP (1:500, Abcam ab13970), rabbit anti-Iba1 (1:500, Wako 019-19741), goat anti-Iba1 (1:500, Abcam ab107159), rat anti-Cd45 FITC (1:200, eBioscience 11-0451-81), rabbit anti-P2ry12 (1:500, from Oleg Butovsky, Harvard Medical School), rabbit anti-cleaved active caspase 3 (1:500, BD Pharmingen 559565), goat anti-Sox9 (1:50, R&D Systems AF3075), mouse anti-NeuN (1:400, Millipore MAB377), and/or goat anti-Vegfr2 (1:200, R&D Sysytems AF644) at 4 °C overnight. Slides were then washed with PBT and exposed to secondary antibody (1:200, Alexa 488 or 555 donkey anti-rabbit IgG, donkey anti-goat IgG, donkey anti-rat IgG, donkey anti-mouse IgG and/or Alexa 488 goat anti-chicken IgG, Life Technologies) for 2 h at RT. Sections were mounted using Aqua Poly/Mount (Polysciences Inc.). Fluorescent IHC images were captured on a Zeiss Axioplan 2 fluorescent microscope. Brightness and/or contrast of the entire image was adjusted using Adobe Photoshop CS5.1 if deemed appropriate.

### Brain tissue culturing and cytokine/chemokine assay

Embryonic wild-type and IUE (E14.5) brains were collected at ~ E14.75 (6 h following IUE) or at E17.5 (3 days following IUE) and placed in a 48-well tissue culture plate (10062-898, VWR) with 300 μl of culture media containing (*v*/*v*) 56.4% DMEM (Gibco 11965-092, Thermo Fisher Scientific), 28.2% F-12 (Gibco 11765-054, Thermo Fisher Scientific), 5% fetal bovine serum, 5% horse serum, 2% B-27 supplement (Gibco 17504-044, Thermo Fisher Scientific), 1% N2 supplement (Gibco 17502-048, Thermo Fisher Scientific), 1% GlutaMAX (Gibco 35050-061, Thermo Fisher Scientific), 1% penicillin/streptomycin (Gibco 15140-148, Thermo Fisher Scientific), and 0.4% fungizone (Gibco 15290-018, Thermo Fisher Scientific). Brains were placed in a 37 °C incubator with 5% CO_2_. For the cytokine/chemokine assay, culture media was collected 6 h following the start of culturing. The collected culture media was spun down at 3000×*g* for 10 min to remove debris, and the middle 200 μl of the culture media was removed and flash frozen with liquid nitrogen. The resulting samples were sent to Eve Technologies (Eve Technologies, University of Calgary) and analyzed using the Mouse Cytokine Array/Chemokine Array 31-PLEX (MD31). Each sample was analyzed for eotaxin, G-CSF, IL-1β, IL-6, IL-10, IP-10, MCP-1, MIP-1α, MIP-1β, MIP-2, RANTES, and TNFα (E14.75 wild-type brain *n* = 3, IUE brain *n* = 3; E17.5 wild-type brain *n* = 3, IUE brain *n* = 4).

### Quantification and statistical analysis

The first four 20-μm sections prior to the start of Fezf1 staining (a hypothalamic marker) were used for all cortex cell counts, and the first four 20-μm Fezf1+ sections were used for all hypothalamic cell counts (*n* = 3–6 from 2 to 4 dams, unless otherwise mentioned). ImageJ was used to determine the relative downregulation of P2ry12 staining between E15.5 wild-type and IUE brains. Quantitative results for all cell counts and cytokine/chemokine protein levels are represented by mean scores ±SEM and were analyzed by two-tailed unpaired *t* tests using Prism 6 (GraphPad Software).

## Results

### In utero electroporation alters microglia morphology and expression signatures

**Fig. 1:**
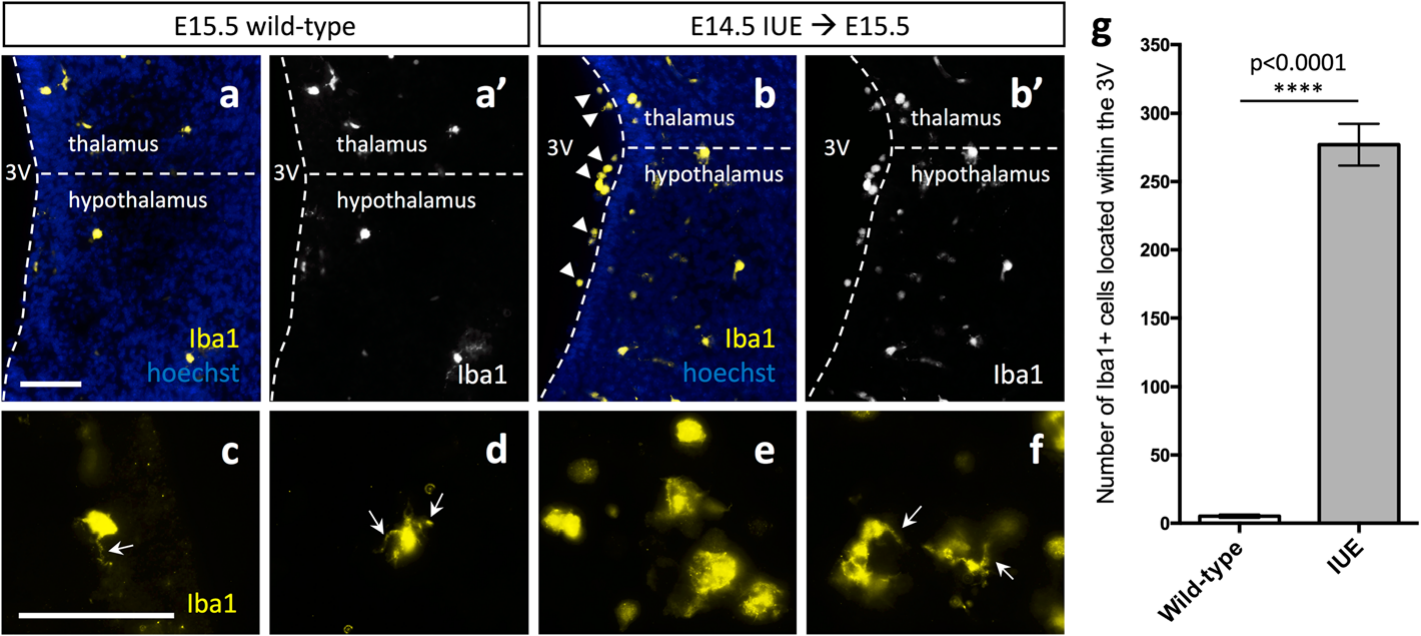
Altered microglia morphology following in utero electroporation. Expression of Iba1 in E15.5 wild-type (**a**, **a’**, **c**, **d**) and E15.5 *pCIG2* IUE (E14.5) brains (**b**, **b’**, **e**, **f**) demonstrate an increase in the number of Iba1+ amoeboid cells lining the third ventricle of IUE brains. 3V, third ventricle. Dashed lines outline the ventricle and mark the division between the thalamus and hypothalamus. White arrows mark Iba1+ microglial projections. Scale bar represents 100 μm (**a**–**f**). **g** Quantification of Iba1+ cells within the third ventricle shows an increase in the number of Iba1+ cells in IUE brains as compared to wild-type brains (mean ± SEM; wild-type *n* = 3, IUE *n* = 3; *p* < 0.0001)

We used the *pCIG2* expression vector, which contains a β-actin promoter/CMV enhancer and an IRES–EGFP cassette, to study the effects of IUE on microglia. *pCIG2* DNA was injected into the lateral ventricle of embryonic day (E) 14.5 brains that were still intact in the uterine horns but temporarily removed from the body cavity of pregnant dams. Any plasmid injected into the lateral ventricle diffuses into the third ventricle given that the ventricles are still connected at this time. Twenty-four hours following the IUE procedure, we stained E15.5 brains for GFP to detect the patch of cells successfully electroporated. GFP-labeled cell bodies and projections were observed lining the lateral ventricles in the cortex (Additional file 1: Figure S1A, arrows), the third ventricle in the hypothalamus (Additional file 1: Figure S1B, arrows), or both (Additional file 1: Figure S1C-D’, arrows). We found that IUE at E14.5 and dissection at E15.5 caused an increase in the number of Iba1+ amoeboid microglia near the third ventricle of the hypothalamus as compared to E15.5 wild-type brains (Fig. 1a–b’). Although we were still able to see some microglial projections in E15.5 IUE (E14.5) brains (Fig. 1f, arrows), there was a noticeable increase in Iba1+ amoeboid microglia (Fig. 1e), especially lining the third ventricle of the hypothalamus (Fig. 1b, arrowhead).

**Fig. 2:**
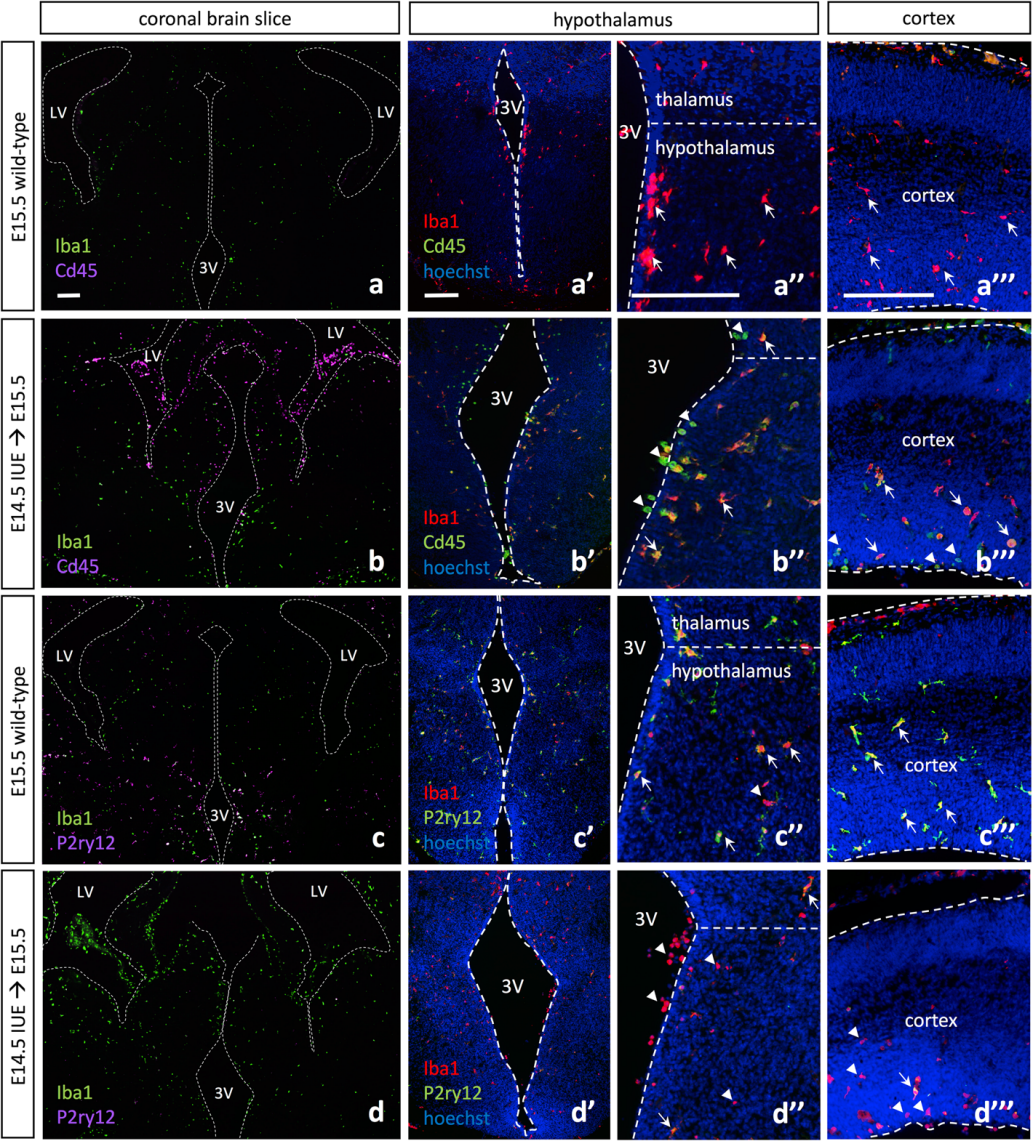
In utero electroporation alters microglia expression signatures throughout the embryonic brain. **a** E15.5 wild-type Iba1 and Cd45 expression show even distribution of Iba1+ cells throughout the brain parenchyma and Cd45^high^ cells lining the periphery of the brain. Higher magnification images of E15.5 wild-type Iba1 and Cd45 expression in the embryonic hypothalamus (**a’**, **a”**) and cortex (**a”’**). **b** E15.5 *pCIG2* IUE (E14.5) Iba1 and Cd45 expression show even distribution of Iba1+ cells throughout the brain parenchyma, while Cd45^high^ cells are present both lining the ventricles in the brain parenchyma and surrounding the periphery of the brain. Higher magnification images of E15.5 *pCIG2* IUE (E14.5) Iba1 and Cd45 expression in the embryonic hypothalamus (**b’**, **b”**) and cortex (**b”’**). **c** E15.5 wild-type Iba1 and P2ry12 expression shows even distribution of both Iba1+ and P2ry12+ cells throughout the brain parenchyma. Higher magnification images of E15.5 wild-type Iba1 and P2ry12 expression in the embryonic hypothalamus (**c’**, **c”**) and cortex (**c”’**). **d** E15.5 *pCIG2* IUE (E14.5) brains highlight Iba1 and P2ry12 expression. Iba1+ cells are evenly distributed throughout the brain parenchyma, while P2ry12 expression is downregulated or absent. Higher magnification images of E15.5 *pCIG2* IUE (E14.5) Iba1 and P2ry12 expression in the embryonic hypothalamus (**d’**, **d”**) and cortex (**d”’**). LV, lateral ventricle; 3V, third ventricle. Dashed lines outline the ventricles and mark the division between the thalamus and hypothalamus. White arrows mark Iba1+ single-positive (**a”**–**a”’**), Iba1+/Cd45^high^ double-positive (**b”**–**b”’**), or Iba1+/P2ry12+ double-positive (**c”**, **c”’**, **d”**, **d”’**) cells, while white arrowheads mark Cd45^high^ single-positive (**b”**–**b”’**), or Iba1+ single-positive (**c”**, **d”**, **d”’**) cells. Scale bar represents 250 μm (**a**–**d”’**)

To better characterize the amoeboid microglia we observed along the third ventricle and within the parenchyma following IUE, we conducted immunohistochemistry with Cd45 and the microglia-specific marker P2ry12. We demonstrated that the amoeboid microglia present in the brain parenchyma displayed altered expression signatures, including the upregulation of Cd45 (Fig. 2a–b”’, arrowheads; Additional file 1: Figure S6) and downregulation or complete absence of the microglia-specific marker P2ry12 (Fig. 2c–d”’, arrowheads). Higher magnification images of Cd45 and P2ry12 expression by hypothalamic wild-type and IUE microglia demonstrated that wild-type microglial projections displayed low levels of Cd45 and high levels of P2ry12 (Additional file 1: Figure S2A, C; arrowheads and arrows), while IUE microglia have lost the majority of their projections and become amoeboid, with the cell surface displaying high levels of Cd45 and lower levels of P2ry12 (Additional file 1: Figure S2B, D; arrowheads and arrows). Quantification of Iba1^+^/Cd45^high^ cells within the hypothalamus (Fig. 4u) and cortex (Fig. 4v) showed a significant increase in Iba1^+^/Cd45^high^ cells within E15.5 IUE (E14.5) brains as compared to wild-type (Fig. 4u, v, hypothalamus *p* < 0.0001, cortex *p* = 0.0023). Similarly, quantification of P2ry12 staining in the hypothalamus (Additional file 1: Figure S3A-B’) and cortex (Additional file 1: Figure S3E-F’) showed a significant decrease in P2ry12 staining within E15.5 IUE (E14.5) brains as compared to wild-type (Additional file 1: Figure S3C-D, G-H; hypothalamus *p* = 0.0018 and *p* = 0.0245; cortex *p* = 0.0004 and *p* = 0.0042). Although altered morphology and changes in expression were observed elsewhere in the brain (Fig. 2a–d), we focused our analysis on the hypothalamus (Fig. 2a’–d’, a”–d”) and cortex (Fig. 2a”’–d”’). Moreover, we observed not only these changes in microglial morphology and expression signature in the cortical and hypothalamic regions in which an IUE GFP+ patch was present (Additional file 1: Figure S4B, D: arrows), but also IUE appeared to affect the contralateral side of the brain (Additional file 1: Figure S4A, C; arrowheads).

In order to determine whether this phenomenon was universal, rather than specific to the *pCIG2* construct and GFP fluorescent protein itself, we also performed IUE with the *pCIC-Ascl1* expression vector, which contains a β-actin promoter/CMV enhancer upstream of the *Ascl1* sequence and an IRES–mCherry cassette, as well as the *pRFP-C-RS* expression vector, which contains a CMV promoter and a tRFP cassette. mCherry- or RFP-labeled cell bodies and projections were observed lining the lateral ventricles in the cortex (Additional file 1: Figure S1E-E’, G-G’; arrows) and/or the third ventricle in the thalamus and hypothalamus (Additional file 1: Figure S1F-F’, H-H’; arrows) following IUE with *pCIC-Ascl1* and *pRFP-C-RS*, respectively. Similar to what was observed following IUE with the *pCIG2* construct, amoeboid microglia were present in the brain parenchyma that displayed altered expression signatures, including the upregulation of Cd45 (Additional file 1: Figure S5A-A”’, C-C”’; arrows) and downregulation or complete absence of P2ry12 (Additional file 1: Figure S5B-B”’, D-D”’; arrowheads). Together, these results suggest that IUE alters both microglia morphology and expression signatures.

### In utero electroporation elicits peripheral macrophage entry into the brain parenchyma

Given the accumulation of Iba1^+^/P2ry12^−^ cells within the ventricles and in the brain parenchyma (Fig. 2d–d”’), we next examined whether these amoeboid cells could be comprised of a mixed population of microglia and macrophages generated from peripheral monocytes that had recently entered the brain parenchyma. Following E14.5 IUE and dissection at E15.5, we observed a significant increase in the number of Iba1+ cells located within the third ventricle adjacent to the hypothalamus (Fig. 1a–b’, g; *p* < 0.0001). Given that macrophages have Fc receptors that bind IgG, and that IgG antibodies are present at a high quantity in the peripheral circulation, cells that have recently entered the brain parenchyma from the periphery often have some IgG bound by their Fc receptors [31–33]. Therefore, Iba1^+^/IgG^+^ cells that were P2ry12^−^ might represent a population that had recently entered the brain from the periphery.

**Fig. 3:**
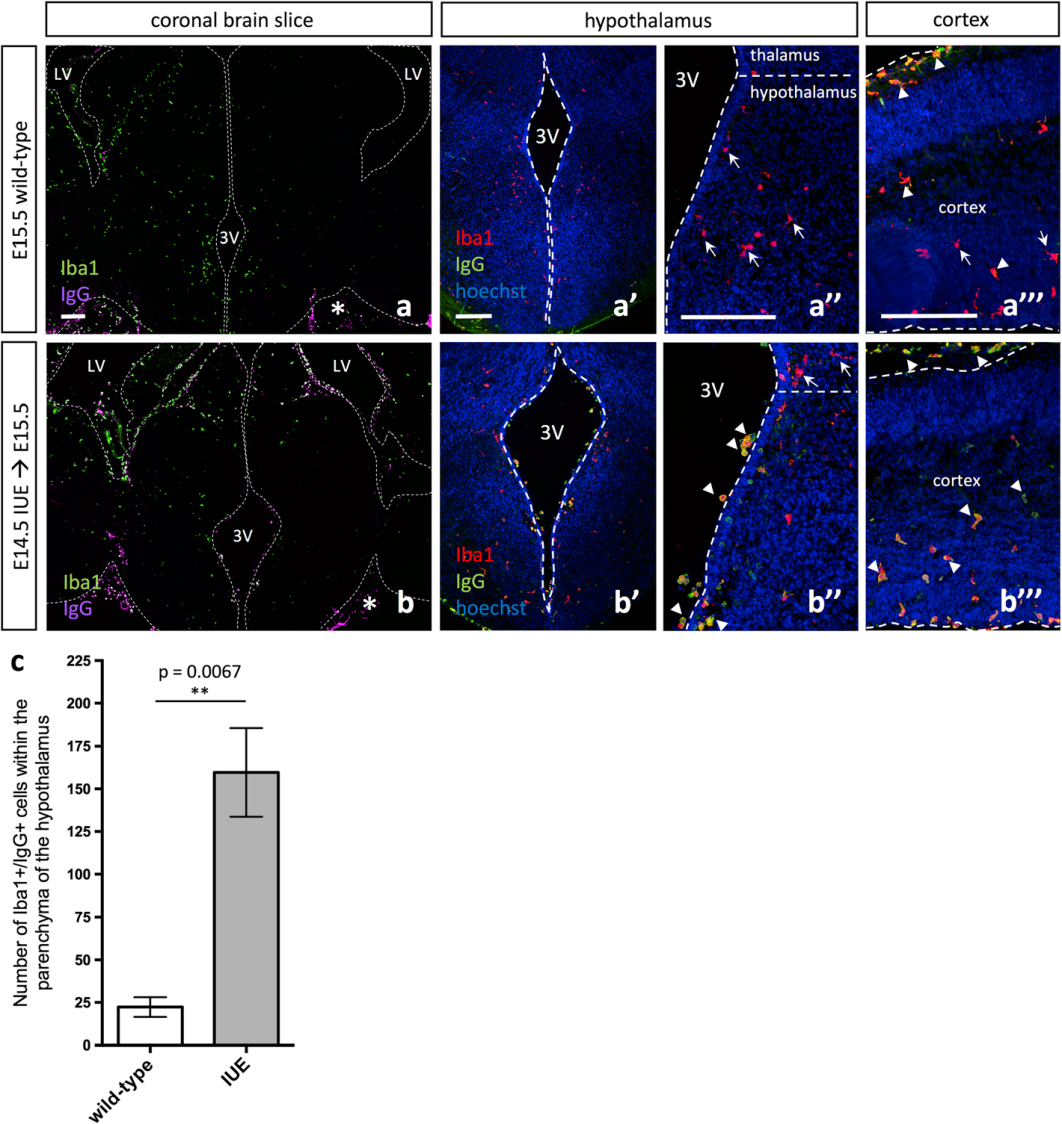
Peripheral macrophages invade the brain parenchyma embryonically following in utero electroporation. **a** E15.5 wild-type Iba1 and IgG expression show even distribution of Iba1+ cells throughout the brain parenchyma and IgG+ cells lining the periphery of the brain (asterisk). Higher magnification images of E15.5 wild-type Iba1 and IgG expression in the embryonic hypothalamus (**a’**, **a”**) and cortex (**a”’**). **b** E15.5 *pCIG2* IUE (E14.5) brains highlight Iba1 and IgG expression. Iba1+ cells are evenly distributed throughout the brain parenchyma, while IgG cells are present both lining the ventricles in the brain parenchyma and surrounding the periphery of the brain (asterisk). Higher magnification images of E15.5 *pCIG2* IUE (E14.5) Iba1 and IgG expression in the embryonic hypothalamus (**b’**, **b”**) and cortex (**b”’**). LV, lateral ventricle; 3V, third ventricle. Dashed lines outline the ventricles and pial surface and mark the division between the thalamus and hypothalamus. White arrows mark Iba1+ single-positive (**a”**, **a”’**, **b”**) cells, and white arrowheads mark Iba1+/IgG+ double-positive (**a”’**, **b”**, **b”’**) cells. Scale bar represents 250 μm (**a**–**b”’**). **c** Quantification of Iba1+/IgG+ cells within the parenchyma of the hypothalamus in IUE brains as compared to wild-type brains (mean ± SEM; wild-type *n* = 3, IUE *n* = 3; *p* = 0.0067)

Immunohistochemistry (IHC) with Iba1 and IgG antibodies demonstrated an increase in Iba1^+^/IgG^+^ double-positive cells within the parenchyma of IUE brains (Fig. 3b–b”’, Additional file 1: Figure S6, arrowhead), while they were only observed lining the periphery of wild-type brains (Fig. 3a–a”’, asterisk). Similar to the observed changes in morphology and expression signatures, Iba1^+^/IgG^+^ accumulation in the brain parenchyma was observed both in the hypothalamus (Fig. 3a’, a”, b’, b”, Additional file 1: Figure S6A-A”) and cortex (Fig. 3a”’, b”’, Additional file 1: Figure S6B-C). Quantification of Iba1^+^/IgG^+^ cells within the hypothalamus showed a significant increase in Iba1^+^/IgG^+^ cells within E15.5 IUE (E14.5) brains as compared to wild-type (Fig. 3c, *p* = 0.0067), consistent with the notion that IUE causes peripheral myeloid cells to invade the developing brain.

### Incorporation of foreign DNA during in utero electroporation is responsible for inducing an immune response

**Fig. 4:**
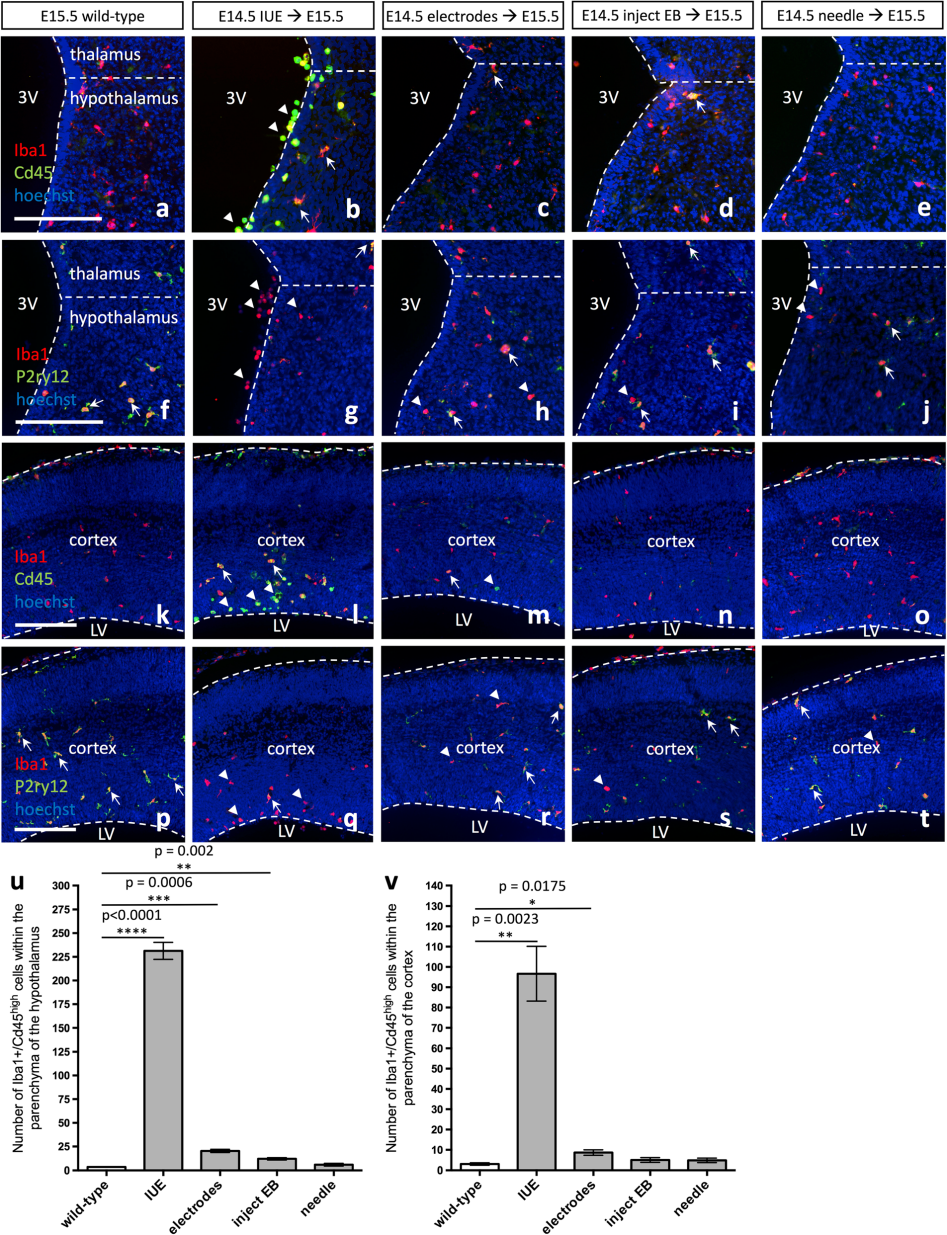
Foreign DNA is responsible for altering microglia morphology and expression signatures in the embryonic brain. Expression of Iba1 and Cd45 in **a**, **k** E15.5 wild-type, **b**, **l** E15.5 *pCIG2* IUE (E14.5), **c**, **m** E15.5 brains electroporated with electrode paddles at E14.5, **d**, **n** E15.5 brains injected with elution buffer (EB) at E14.5, or **e**, **o** E15.5 brains stabbed with a sterile glass needle at E14.5. Expression of Iba1 and P2ry12 in **f**, **p** E15.5 wild-type, **g**, **q** E15.5 *pCIG2* IUE (E14.5), **h**, **r** E15.5 brains electroporated with electrode paddles at E14.5, **i**, **s** E15.5 brains injected with EB at E14.5, or **j**, **t** E15.5 brains stabbed with a sterile glass needle at E14.5. Hypothalamus (**a**–**j**) and cortex (**k**–**t**). 3V, third ventricle; LV, lateral ventricle. Dashed lines outline the ventricles and mark the division between the thalamus and hypothalamus. White arrows mark Iba1+/Cd45^high^ double-positive (**b**–**d**, **l**–**m**), or Iba1+/P2ry12+ double-positive (**f**–**j**, **p**–**t**) cells, while white arrowheads mark Cd45^high^ single-positive (**b**, **l**–**m**), or Iba1+ single-positive (**g**–**j**, **q**–**t**) cells. Scale bar represents 250 μm (**a**–**t**). **u** Quantification of Iba1+/Cd45^high^ cells within the parenchyma of the hypothalamus in wild-type brains as compared to IUE brains and the other treatments (mean ± SEM; wild-type *n* = 3; IUE *n* = 3, *p* < 0.0001; electrodes *n* = 3, *p* = 0.0006; inject EB *n* = 3, *p* = 0.002; needle *n* = 4, *p* = 0.2194). **v** Quantification of Iba1+/Cd45^high^ cells within the parenchyma of the cortex in wild-type brains as compared to IUE brains and the other treatments (mean ± SEM; wild-type *n* = 3; IUE *n* = 3, *p* = 0.0023; electrodes *n* = 3, *p* = 0.0175; inject EB *n* = 3, *p* = 0.1963; needle *n* = 4, *p* = 0.2660)

Given the marked changes in microglia expression signatures and morphology observed following IUE, we next sought to determine what aspect of the IUE procedure was sufficient to induce such a striking immune response; namely, whether it was the injection through neural tissue, the shock of the electroporation itself, or the expression of foreign DNA. Conceivably, any of these steps could lead to microglia activation. To test this question, we compared E15.5 wild-type brains to (1) E15.5 *pCIG2* IUE (E14.5) brains, (2) E15.5 brains electroporated with electrode paddles only (at E14.5), (3) E15.5 brains injected with elution buffer (EB) only (at E14.5), or (4) E15.5 brains punctured with the glass needle (at E14.5). We detected a significant increase in the number of Iba1^+^ cells that upregulated Cd45 in the hypothalamus of E15.5 brains electroporated with paddles alone (Fig. 4c, arrow; Fig. 4u, *p* = 0.0006) and injected with EB alone (Fig. 4d, arrow; Fig. 4u, *p* = 0.002); however, brains that received the entire IUE procedure showed a striking increase in Cd45 expression in the hypothalamus (Fig. 4b, arrows; Fig. 4u, *p* < 0.0001). At the same time, a decrease in the microglia-specific marker P2ry12 was observed in Iba1+ cells, as compared to wild-type (Fig. 4f–j, arrowhead). In the cortex, the only treatment that resulted in a significant increase in the number of Iba1^+^ cells that upregulated Cd45, besides the entire IUE procedure (Fig. 4l, arrow; Fig. 4v, *p* = 0.0023), was the E15.5 brain electroporated with electrode paddles at E14.5 (Fig. 4m, arrow; Fig. 4v, *p* = 0.0175). Furthermore, P2ry12 was similarly downregulated in Iba1+ cells in the cortex (Fig. 4p–t, arrowhead).

In order to further investigate the exact aspect of the IUE procedure that was causing such a noticeable immune response, we employed milder IUE parameters. Specifically, we injected *pCIG2* DNA at a concentration of 0.5 μg/μL into the lateral ventricle of E14.5 brains, which were pulsed using 30 V (50 ms) four times, separated by intervals of 1000 ms (versus 45 V (50 ms) five times separated by intervals of 950 ms, as used previously). Similar to what we observed using the original IUE parameters, IUE with the gentler parameters still resulted in the upregulation of Cd45 (Additional file 1: Figure S7A-A”’, arrowheads) and downregulation or loss of P2ry12 (Additional file 1: Figure S7B-B”’, arrowheads). Furthermore, we also performed the entire IUE procedure using HEPES in place of DNA, in order to analyze the effect of drawing up a negatively charged substance during IUE to test if the immune response was due to foreign DNA or simply the negative charge of DNA; however, this did not result in changes to Cd45 or P2ry12 expression levels (Additional file 1: Figure S7C-D”’, arrows). Taken together, the results suggest that the uptake and incorporation of foreign DNA during the IUE procedure is what ultimately appears to induce an immune response.

### In utero electroporation results in increased cell death in the hypothalamus

**Fig. 5:**
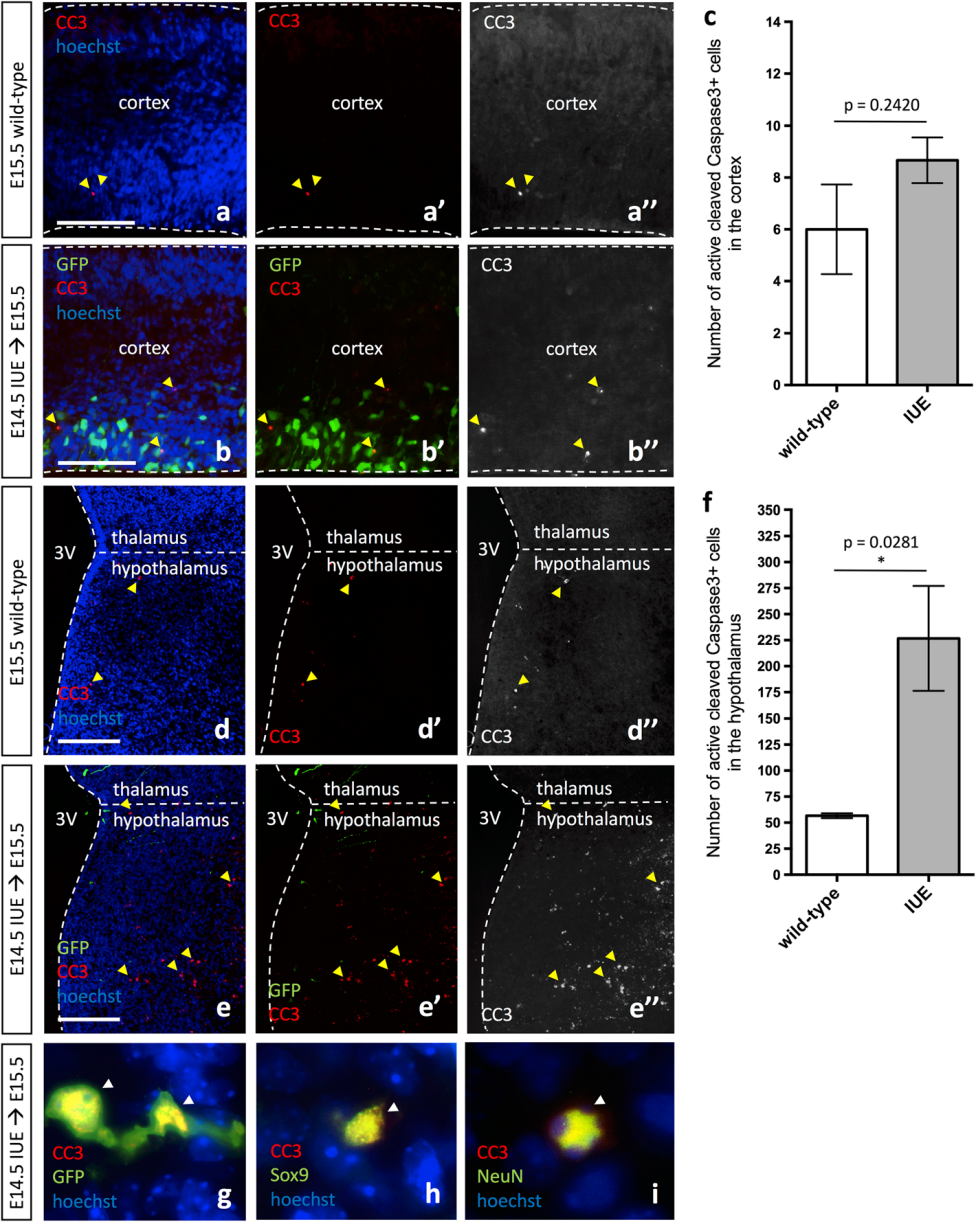
In utero electroporation induces cell death in the developing hypothalamus but not the cortex. Expression of GFP and/or active cleaved caspase 3 (CC3) in E15.5 wild-type (**a**–**a”**, **d**–**d”**) and *pCIG2* IUE (E14.5) brains (**b**–**b”**, **e**–**e”**) highlights the increase in cell death in the hypothalamus of IUE brains (**e**–**e”**). Cortex (**a**–**b”**) and hypothalamus (**d**–**e”**). 3V, third ventricle. Dashed lines outline the ventricles and mark the division between the thalamus and hypothalamus. Yellow arrowheads mark CC3+ cells. Scale bar represents 250 μm (**a**–**e”**). **c** Quantification of CC3+ cells in the cortex does not show a significant increase in the number of CC3+ cells in IUE brains as compared to wild-type brains (mean ± S.E.M; wild-type *n* = 3, IUE *n* = 3; *p* = 0.2420). **f** Quantification of CC3+ cells in the hypothalamus shows an increase in the number of CC3+ cells in IUE brains as compared to wild-type brains (mean ± S.E.M; wild-type *n* = 3, IUE *n* = 3; *p* = 0.0281). **g** GFP+/CC3+ double-positive hypothalamic radial glia. **h** Sox9+/CC3+ double-positive hypothalamic cell. **i** NeuN+/CC3+ double-positive hypothalamic neuron. While arrowheads mark CC3+ cells

Considering the marked cellular alterations in the microglial profile following IUE, we next assayed whether ectopic activation of microglia had effects on nearby cells in the developing brain. Specifically, we assayed for increased apoptosis following IUE in the cortex and hypothalamus. We observed no effect of IUE on apoptosis in the cortex (Fig. 5a–c, arrowhead; *p* = 0.2420), consistent with previous results [34]. In the hypothalamus, however, we observed a significant increase in the number of anti-active cleaved caspase 3+ (CC3) cells in IUE brains as compared to wild-type brains (Fig. 5d–f, arrowhead; *p* = 0.0281). While the majority of the CC3+ cells within the hypothalamus appeared to be apoptotic bodies (Fig. 5e”), infrequently we also observed IUE GFP+ radial glia lining the ventricle undergoing cell death (Fig. 5g, arrowhead), in addition to the isolated Sox9+/CC3+ (Fig. 5h, arrowhead) or NeuN+/CC3+ (Fig. 5i, arrowhead) double-positive cell. Moreover, examination of the IUE brain outside of the cortex and hypothalamus demonstrated that the thalamus (2/3 analyzed) and amygdala (1/3 analyzed) of IUE embryos also showed elevated CC3 levels when compared to wild-type embryos (Additional file 1: Figure S8A-D, arrowheads), however, these findings were not consistent between all the IUE brains analyzed for reasons that are not yet obvious. Taken together, the results indicate that while both the cortex and hypothalamus display an immune response following IUE, unlike the cortex, the hypothalamus is more sensitive to IUE and reacts by upregulating cell death programs.

### In utero electroporation elevates pro-inflammatory cytokine and chemokine levels

**Fig. 6:**
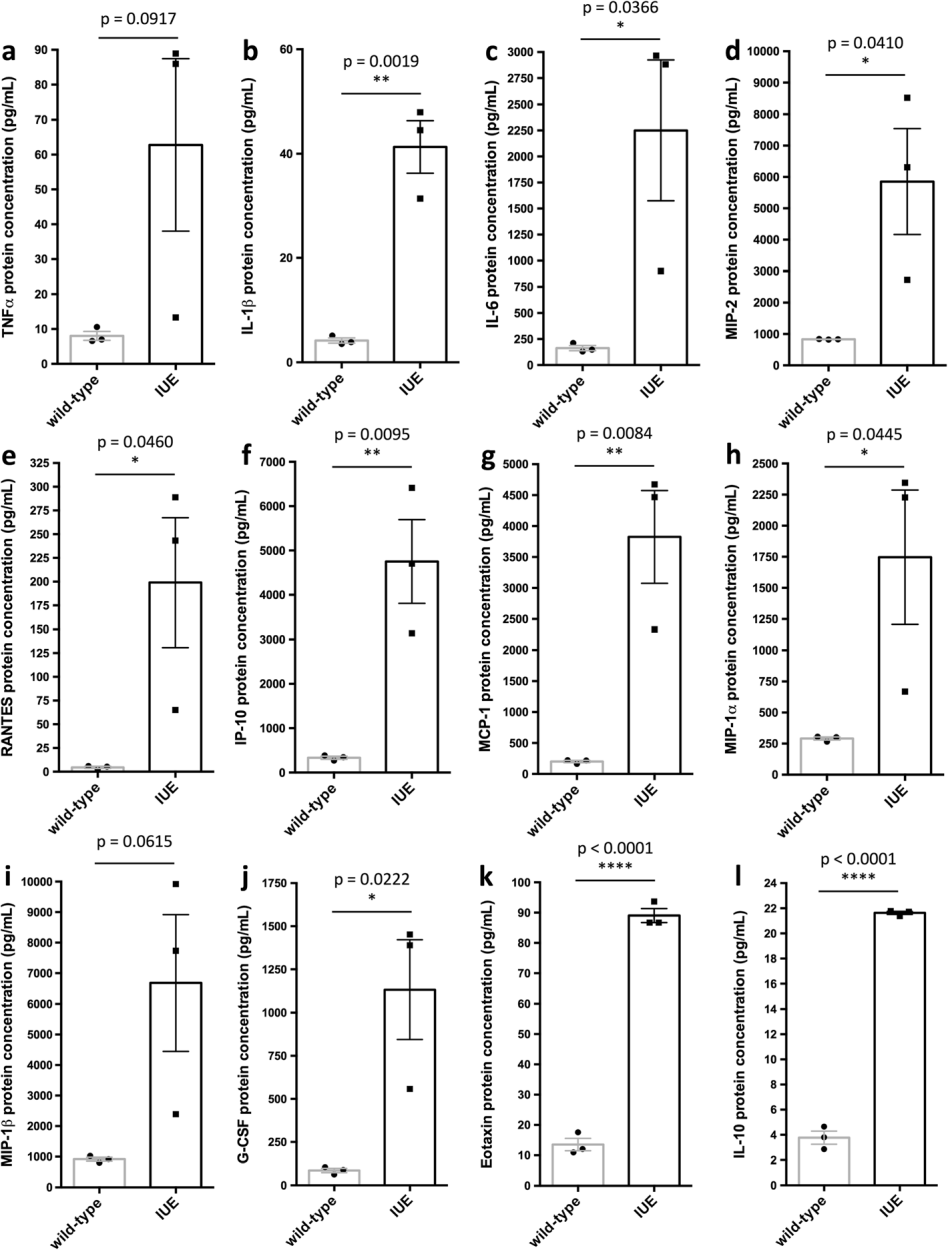
Inflammatory cytokines and chemokines are upregulated in the embryonic brain following in utero electroporation. Cytokine and chemokine assays show an upregulation of **a** tumor necrosis factor alpha (TNFα; *p* = 0.0917), **b** interleukin 1 beta (IL-1β; *p* = 0.0019), **c** interleukin 6 (IL-6; *p* = 0.0366), **d** macrophage inflammatory protein 2 (MIP-2; *p* = 0.0410), **e** regulated upon activation normal T cell expressed and secreted (RANTES, also known as CCL5; *p* = 0.0460), **f** interferon gamma-induced protein 10 (IP-10, also known as CXCL10; *p* = 0.0095), **g** monocyte chemotactic protein 1 (MCP-1, also known as CCL2; *p* = 0.0084), **h** macrophage inflammatory protein 1 alpha (MIP-1α, also known as CCL3; *p* = 0.0445), **i** macrophage inflammatory protein 1 beta (MIP-1β, also known as CCL4; *p* = 0.0615), **j** ganulocyte colony-stimulating factor (G-CSF; *p* = 0.0222), **k** eotaxin (p < 0.0001), and **l** interleukin 10 (IL-10; *p* < 0.0001) in ~ E14.75–15 IUE (E14.5) brains as compared to wild-type (protein levels pg/mL; mean ± S.E.M.; wild-type *n* = 3, IUE *n* = 3)

Given that IUE alters microglia morphology and expression signatures in a way that resembles activation of microglia during a pro-inflammatory immune response, we next measured and compared cytokine and chemokine levels in wild-type and IUE brains. To do this, we cultured whole ~ E14.75 wild-type and IUE (E14.5) brains for 6 h and collected the conditioned media to analyze cytokine and chemokine protein levels. Specifically, we found an upregulation of the pro-inflammatory cytokines and chemokines: tumor necrosis factor alpha (TNFα; Fig. 6a, *p* = 0.0917), interleukin 1 beta (IL-1β; Fig. 6b, *p* = 0.0019), and interleukin 6 (IL-6; Fig. 6c, *p* = 0.0366), as well macrophage inflammatory protein 2 (MIP-2; Fig. 6d, *p* = 0.0410), regulated upon activation normal T cell expressed and secreted (RANTES, also known as CCL5; Fig. 6e, *p* = 0.0460), and interferon gamma-induced protein 10 (IP-10, also known as CXCL10; Fig. 6f, *p* = 0.0095) in IUE brains as compared to wild-type brains. IUE brains also showed elevated levels of monocyte chemotactic protein 1 (MCP-1, also known as CCL2; Fig. 6g, *p* = 0.0084), macrophage inflammatory protein 1 alpha (MIP-1α, also known as CCL3; Fig. 6h, *p* = 0.0445), macrophage inflammatory protein 1 beta (MIP-1β, also known as CCL4; Fig. 6i, *p* = 0.0615), ganulocyte colony-stimulating factor (G-CSF; Fig. 6j, *p* = 0.0222), and eotaxin (Fig. 6k, *p* < 0.0001) as compared to wild-type brains. Together, the results suggest that IUE induces a pro-inflammatory response, which is supported by the changes in microglia morphology and expression signatures, in addition to the infiltration of peripheral myeloid cells.

**Fig. 7:**
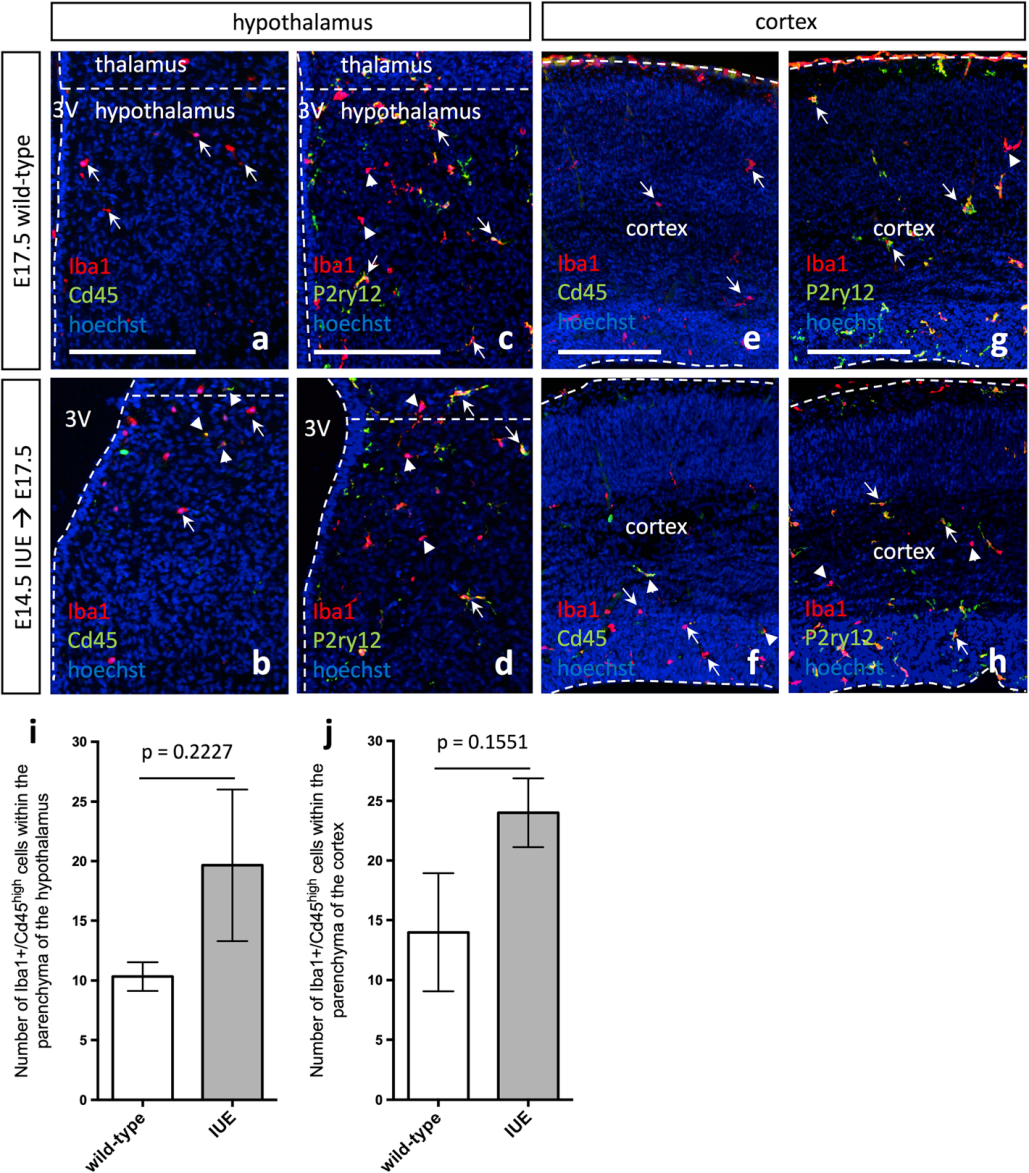
Microglia morphology and expression signatures are comparable to wild-type 3 days following in utero electroporation. **a**, **e** E17.5 wild-type Iba1 and Cd45 expression in the hypothalamus (**a**) and cortex (**e**) show an even distribution of Iba1+ cells throughout the brain parenchyma. **b**, **f** E17.5 *pCIG2* IUE (E14.5) Iba1 and Cd45 expression in the hypothalamus (**b**) and cortex (**f**) show Iba1+ cells are evenly distributed throughout the brain parenchyma, while very few Cd45^high^ cells are present in the brain parenchyma. **c**, **g** E17.5 wild-type Iba1 and P2ry12 expression in the hypothalamus (**c**) and cortex (**g**) show even distribution of both Iba1+ and P2ry12+ cells throughout the brain parenchyma. **d**, **h** E17.5 *pCIG2* IUE (E14.5) Iba1 and P2ry12 expression in the hypothalamus (**d**) and cortex (**h**) show Iba1+ and P2ry12+ cells are relatively evenly distributed throughout the brain parenchyma. Scale bar represents 250 μm (a-e”). 3V, third ventricle. Dashed lines outline the ventricles and mark the division between the thalamus and hypothalamus. White arrows mark Iba1+ single-positive (**a**–**b**, **e**–**f**) or Iba1+/P2ry12+ double-positive (**c**–**d**, **g**–**h**) cells, while white arrowheads mark Iba1+/Cd45^high^ double-positive (**b**, **f**) or Iba1+ single-positive (**c**–**d**, **g**–**h**) cells. **i** Quantification of Iba1+/Cd45^high^ cells within the parenchyma of the hypothalamus in IUE brains as compared to wild-type (mean ± SEM; wild-type *n* = 3, IUE *n* = 3; *p* = 0.2227). **j** Quantification of Iba1+/Cd45^high^ cells within the parenchyma of the cortex in IUE brains as compared to wild-type (mean ± SEM; wild-type *n* = 3, IUE *n* = 3; *p* = 0.1551)

Given the upregulation of the anti-inflammatory cytokine interleukin 10 in IUE brains (IL-10; Fig. 6l, *p* < 0.0001), which can be produced by activated macrophages in order to suppress macrophage activation and the further production of pro-inflammatory cytokines/chemokines [35, 36], next, we decided to determine for how long the inflammatory response and developmental alterations resulting from IUE persisted. IHC for Cd45 and P2ry12 in both the hypothalamus and cortex demonstrated a decrease in Cd45 and a normalization of P2ry12 expression in IUE brains after approximately 3 days (E17.5; Fig. 7a–h, arrowhead and arrows). Quantification of Iba1^+^/Cd45^high^ cells within the parenchyma of the hypothalamus (Fig. 7i, *p* = 0.2227) and cortex (Fig. 7j, *p* = 0.1551) showed that E17.5 IUE brains and wild-type brains were comparable by E17.5. Although microglia morphology and expression signatures appeared to recover in E17.5 IUE (E14.5) brains (Fig. 7), we also measured cytokine and chemokine levels in E17.5 IUE (E14.5) brains to determine if these secreted factors also returned to baseline. For all cytokine and chemokine measured in E17.5 IUE (E14.5) brains, none were significantly different than the levels observed in wild-type brains. At the same time, we did notice considerable variation in cytokine and chemokine levels across the IUE E17.5 brains, resulting in a trend towards a maintained increase but that collectively did not reach significance. Specifically, TNFα (Additional file 1: Figure S9A, *p* = 0.0942), IL-1β (Additional file 1: Figure S9B, *p* = 0.1584), and IL-6 (Additional file 1: Figure S9C, *p* = 0.3289), as well as MIP-2 (Additional file 1: Figure S9D, *p* = 0.1703), RANTES (Additional file 1: Figure S9E, *p* = 0.1981), and IP-10 (Additional file 1: Figure S9F, *p* = 0.0527), were all trending towards higher levels in IUE brains. Similarly, some E17.5 IUE (E14.5) brains also showed elevated levels of MCP-1 (Additional file 1: Figure S9G, *p* = 0.4466), MIP-1α (Additional file 1: Figure S9H, *p* = 0.4887), MIP-1β (Additional file 1: Figure S9I, *p* = 0.3008), G-CSF (Additional file 1: Figure S9J, *p* = 0.3193), and IL-10 (Additional file 1: Figure S9L, *p* = 0.3789) but again, not at the level of significance. In fact, eotaxin (Additional file 1: Figure S9K, *p* = 0.9152) was the only marker that had normalized fully to levels observed in wild-type brains. Together, the results suggest that IUE induces an immediate pro-inflammatory response, which appears to recover at different rates in different embryos.

## Discussion

Here, we used IUE and the *pCIG2*, *pCIC-Ascl*, and *pRFP-C-RS* expression vectors to study the effects of this technique on microglia in the embryonic brain. Within 24 h following the IUE procedure, we observed an increase in amoeboid microglia that displayed altered expression signatures, including the upregulation of Cd45 and downregulation of P2ry12. IUE universally elevated pro-inflammatory cytokine and chemokine levels throughout the embryonic brain and induced a significant increase in cell death in the developing hypothalamus. The changes in microglial morphology and expression signatures apparent in IUE brains return to a state comparable to wild-type brains around 3 days following the IUE procedure. Although this time to normalization is only 3 days, which is perhaps not unexpected given the immature nature of the embryonic immune system, compiled together, the results suggest that there is the potential for immune-related damage to the embryonic brain during the first 3 days following IUE, particularly in the developing hypothalamus.

Given that microglia originate from yolk sac-derived Cd45^high^ macrophages, microglia are themselves phagocytic cells with the potential to upregulate Cd45 and downregulate P2ry12 during times of inflammation [37]. However, since we also observed numerous P2ry12^−^ cells lining the ventricles in the brain parenchyma that were Iba1^+^IgG^+^ double-positive, these Cd45^high^/P2ry12^low^ and Cd45^high^/P2ry12^−^ amoeboid cells within the brain of IUE embryos likely represent a mixed population of activated resident microglia and peripheral macrophages, which have recently entered the brain proper [31–33, 37]. This invasion is consistent with how one would predict the embryonic brain to react following IUE, since IUE involves the incorporation of foreign DNA that can trigger an immune response [38–40]. For example, during an immune reaction, the blood-brain barrier (BBB) can become disrupted, thereby allowing monocyte-derived macrophages and other immune cells from the peripheral circulation to enter the brain parenchyma [41–45]. Resident microglia themselves also play an important role during inflammation and have been shown to become activated both in the case of bacterial (e.g., lipopolysaccharide, LPS) and viral (e.g., adenovirus) infection [46, 47]. It will be interesting to determine if embryonic microglia clear the GFP^+^ cells (e.g., cells that contain foreign DNA) following IUE, and if so, whether embryonic microglia respond to the transfected cells using toll-like receptors (TLRs), TAM receptors (Tyro3, Axl, Mer), or other mechanisms shown to be used to recognize viral DNA or foreign cytoplasmic DNA [38–40, 46–48].

Considering that IUE involves both the uptake of foreign DNA and the generation of a foreign protein (e.g., GFP, mCherry, RFP), future experimentation should also determine whether it is the presence of a foreign DNA plasmid or the generation of a foreign protein that is causing the cells in the CNS to mount an immune response following IUE. Given that fluorescent proteins are expressed in a number of transgenic mouse lines, in which we have not observed an immune response (data not shown), we propose it is the uptake and presence of foreign DNA itself in the cell’s cytoplasm that is triggering the immune response we observe following IUE. Moreover, considering that most IUE experimental procedures use constructs coding for a fluorescent protein to label the transfected cells, the current study has demonstrated that careful controls are needed when using this approach. Nonetheless, further investigation of whether the foreign DNA is in fact stimulating the neuroimmune response following IUE is needed, as well as a deeper appreciation of the consequences of the resulting immune response.

During microglia activation and the subsequent infiltration of peripheral myeloid cells, inflammatory cytokine and chemokine levels are often elevated [46, 47, 49, 50]. Consistent with an immune response, within 6 h following IUE, we observed increases in both pro-inflammatory cytokines and chemokines: TNFα, IL-1β, IL-6, MIP-2, RANTES (CCL5), IP-10 (CXCL10), MCP-1 (CCL2), MIP-1α (CCL3), MIP-1β (CCL4), G-CSF, and eotaxin. The significantly elevated cytokines and chemokines observed in the media of cultured whole IUE brains imply a universal neuroimmune response, which is consistent with the universal brain-wide activation of microglia we observed. While it might be surprising that the neuroimmune response is not localized to the region where the IUE-positive patches are observed, this is in line with other work showing that cytokines and chemokines appear to signal along neuronal projections and use extracellular diffusion across the vasculature/BBB to signal through the blood and cerebrospinal fluid (CSF) to other brain regions (reviewed in [51, 52]). Moreover, we are examining the effects of IUE in an embryonic brain in which the BBB is just being established and in regions where the BBB is known to be leaky [53], which further supports the easy movement of these cytokines and chemokines throughout the brain parenchyma, blood vessels/bloodstream, and into the CSF. While these pro-inflammatory cytokines and chemokines appear to normalize in some animals by 3 days following IUE, other embryos still displayed elevated cytokine and chemokine levels at E17.5. Interestingly, these pro-inflammatory signals have been reported to be involved in the development of autism spectrum disorders, schizophrenia, and anxiety or depression-related behaviors [14, 54–58]. Given that we show here that IUE itself can induce an immune response and an elevation in pro-inflammatory cytokines/chemokines in the embryonic brain, it is possible that IUE may alter normal developmental programs. Therefore, researchers may want to consider the potential influence of microglia activation when interpreting results obtained following IUE. Of course, further studies are needed to determine if an immune response induced by the expression of foreign DNA following IUE embryonically indeed leads to permanent consequences on normal brain development, wiring, or behavioral outcomes. Together, these results suggest that caution might need to be exercised when employing IUE to label cells in the developing brain, especially if your scientific questions involve short-term time points. Given that the inflammatory environment seems to diminish within 3 days post-IUE—but cautiously not in all animals—it is unclear whether this immune response is transient or whether it has lasting effects on neural development.

Along these lines, we did observe an increase in apoptosis specifically in the hypothalamus, suggesting that at least in this brain region, IUE might permanently affect development. During a pro-inflammatory state, cell death programs are often initiated in order to instigate phagocytic cells such as macrophages and microglia to clear foreign materials. Thus, observing an increase in apoptotic cells within the hypothalamus following IUE suggests that this brain region is particularly sensitive to IUE and inflammation during development. Furthermore, considering inflammation during development can have long-term consequences on learning and memory as well as other behavioral alterations [13–19], we propose that future long-term analyses of animals exposed to IUE during development should include a fluorescent gene control (e.g., *pCIG2*) in addition to a wild-type control, to enable comparison for any long-term consequences of the IUE procedure itself.

## Conclusions

By exploring the effect of IUE on microglia, we demonstrated that *pCIG2*, *pCIC-Ascl1*, or *pRFP-C-RS* IUE alone activates microglia and alters microglial expression signatures. Importantly, IUE was shown to induce cell death in the developing hypothalamus but not the cortex. Moreover, we observed universal upregulation of pro-inflammatory cytokines and chemokines throughout the embryonic brain, consistent with the notion that IUE causes inflammation. These findings demonstrate that IUE might have the unintended consequence of activating microglia in the embryonic brain, which could have long-term consequences in a region-specific manner, particularly within the hypothalamus.

## Additional file

Additional file 1: Supplementary Figures 1–9. (PDF 31554 kb)

## Abbreviations

BBB: Blood-brain barrier
CC3: Cleaved caspase 3
Cd45: Cluster of differentiation 45
CNS: Central nervous system
CSF: Cerebrospinal fluid
DNA: Deoxyribonucleic acid
EMPs: Erythro-myeloid progenitors
G-CSF: Ganulocyte colony-stimulating factor
GFP: Green fluorescent protein
Iba1: Ionized calcium-binding adapter molecule 1
IgG: Immunoglobulin G
IHC: Immunohistochemistry
IL-1β: Interleukin 1 beta
IL-6: Interleukin 6
IP-10: Interferon gamma-induced protein 10
IUE: In utero electroporation
LPS: Lipopolysaccharide
MCP-1: Monocyte chemotactic protein 1
MIP-1α: Macrophage inflammatory protein 1 alpha
MIP-1β: Macrophage inflammatory protein 1 beta
MIP-2: Macrophage inflammatory protein 2
P2ry12: Purinergic receptor P2Y G-protein coupled 12
PBS: Phosphate-buffered saline
PFA: Paraformaldehyde
RANTES: Regulated upon activation normal T cell expressed and secreted
RFP: Red fluorescent protein
RT: Room temperature
Sox9: SRY-Box 9
TLRs: Toll-like receptors
TNFα: Tumor necrosis factor alpha
