# Supplementary material for: In utero electroporation induces cell death and alters embryonic microglia morphology and expression signatures in the developing hypothalamus
Source: J Neuroinflammation. 2018 Jun 12;15:181. doi: 10.1186/s12974-018-1213-6 (PMC5998590; doi:10.1186/s12974-018-1213-6)
Supplement: Supplementary file 1 — Supplementary Figures 1–9. (PDF 31554 kb) [file 12974_2018_1213_MOESM1_ESM.pdf]

Supplementary Materials

Rosin et al, Figure S1

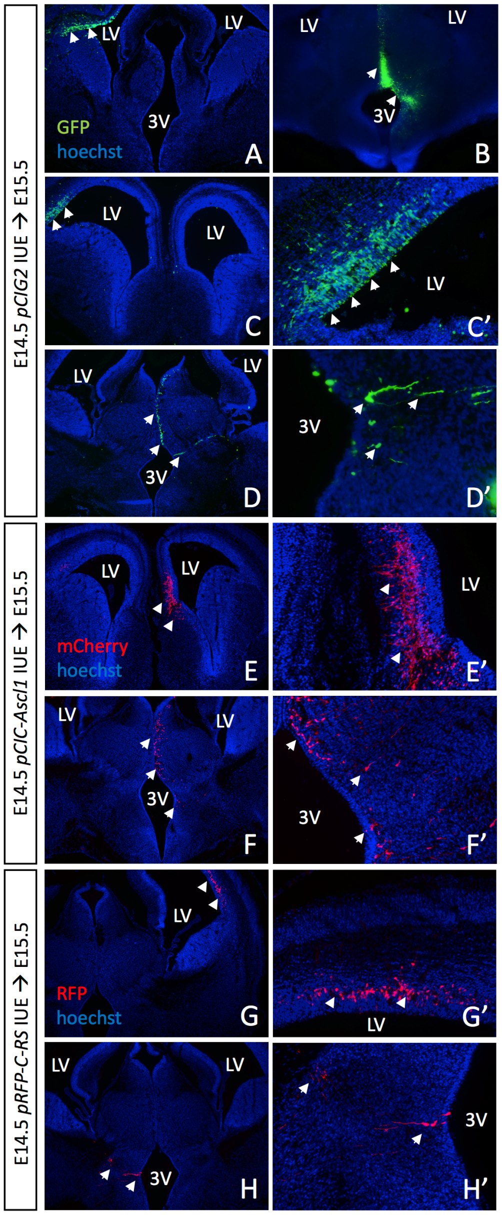

Supplementary Figure 1. Regional expression of *pCIG2, pCIC-Ascl and pRFP-C-RS* patches in the embryonic brain following *in utero* electroporation. Examples of E15.5 *pCIG2* (A-D'), *pCIC-Ascl1* (E-F') and *pFRP-C-RS* (G-H') IUE (E14.5) highlight fluorescent patches in the cortex (A, E-E', G-G'), thalamus/hypothalamus (B, F-F', H-H'), or both the cortex and thalamus/hypothalamus (C-D') following IUE. LV, lateral ventricle; 3V, third ventricle. White arrows mark GFP+, mCherry+ and RFP+ cells.

Rosin et al, Figure S2

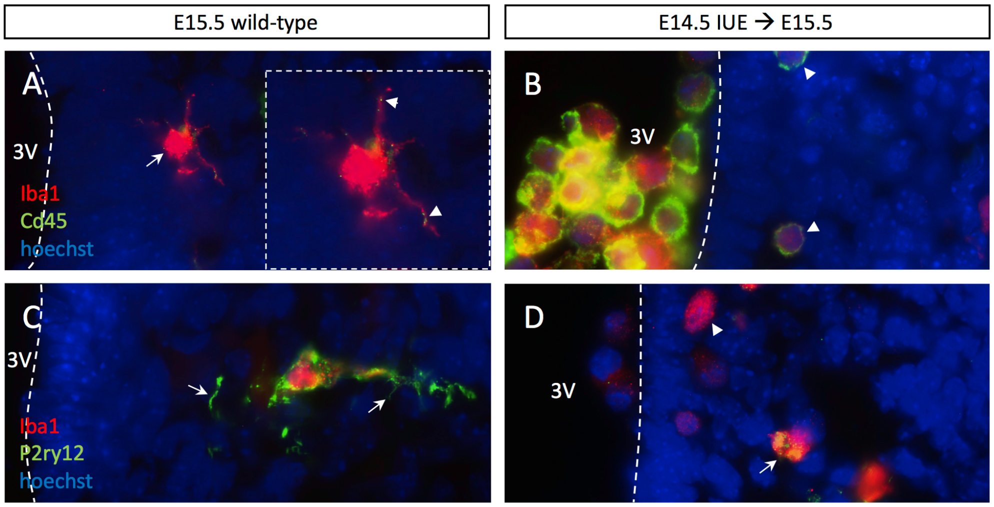

Supplementary Figure 2. ***In utero*** **electroporation alters microglia morphology and expression signatures.** E15.5 hypothalamic Iba1 and Cd45 expression in wild-type (A) and *pCIG2* IUE (E14.5) (B) embryos shows low levels of Cd45 on wild-type Iba1+ microglial projections (A), with higher levels of Cd45 apparent around the entire cell surface of Iba1+ amoeboid microglia following IUE (B). E15.5 hypothalamic Iba1 and P2ry12 expression in wild-type (C) and *pCIG2* IUE (E14.5) (D) embryos shows P2ry12 lining wild-type Iba1+ microglial projections (C), while amoeboid Iba1+ microglia present in IUE brains show little or no P2ry12 (D). 3V, third ventricle. Dashed-lines outline the third ventricle. White arrows mark Iba1+ (A), or Iba1+/P2ry12+ double-positive (C, D) cells, while white arrowheads mark Iba1+/Cd45^{high} (B), or Iba1+ single-positive (D) cells.

Rosin et al, Figure S3

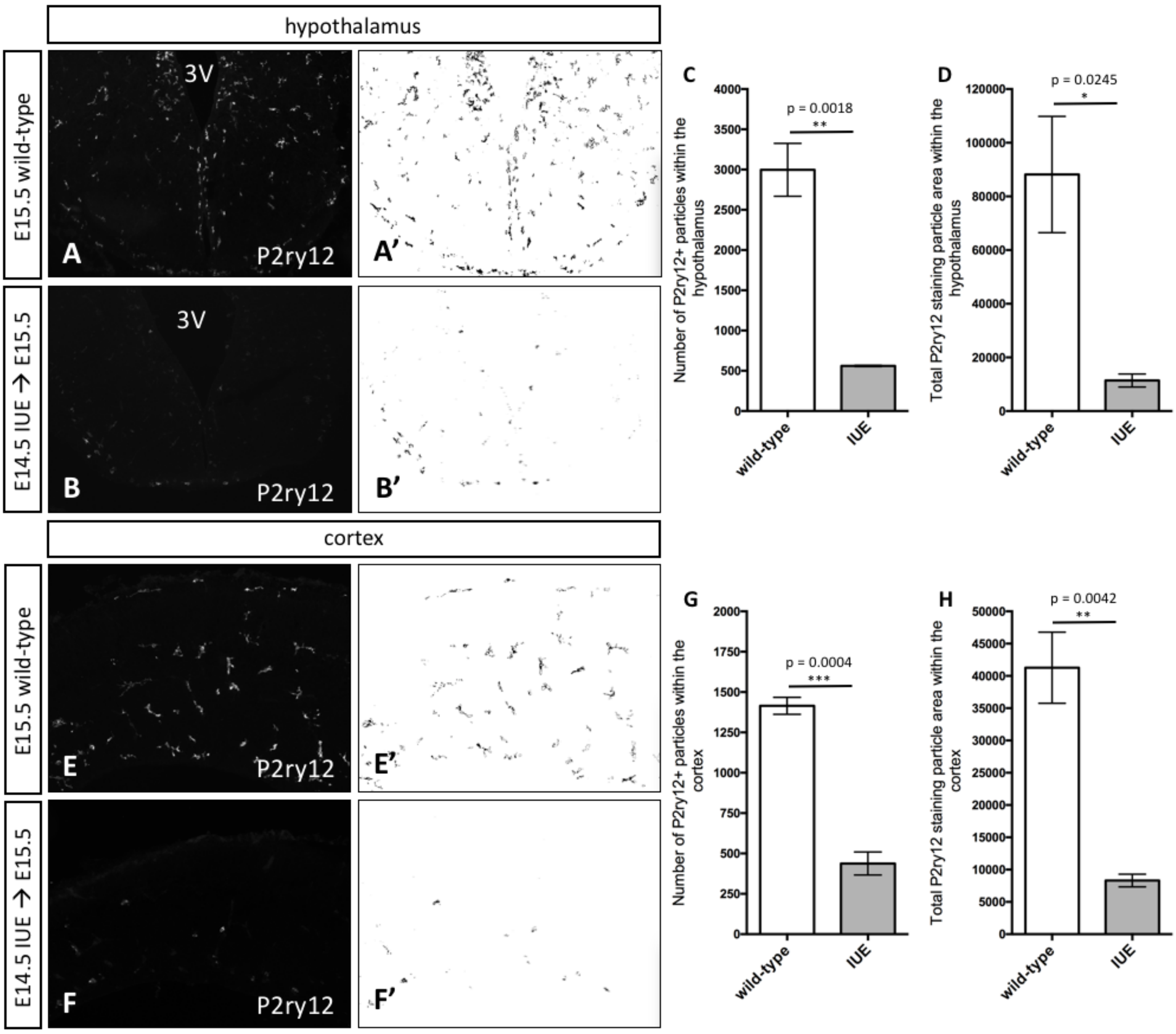

Supplementary Figure 3. *In utero* electroporation decreases P2ry12 expression in microglia throughout the embryonic brain. (A, E) E15.5 wild-type P2ry12 expression in the hypothalamus (A) and cortex (E) shows even distribution of P2ry12+ cells throughout the brain parenchyma. (A', E') Representative E15.5 wild-type ImageJ P2ry12 staining pixel area in the hypothalamus (A') and cortex (E'). (B, F) E15.5 *pCIG2-EGFP* IUE (E14.5) P2ry12 expression in the hypothalamus (B) and cortex (F) shows downregulation or loss of P2ry12 expression. (B', F') Representative E15.5 *pCIG2-EGFP* IUE (E14.5) ImageJ P2ry12 staining pixel area in the hypothalamus (B') and cortex (F'). (C-D) Quantification of P2ry12+ particles (C, $p = 0.0018$) and P2ry12 particle area (D, $p = 0.0245$) within the hypothalamus in IUE brains as compared to wild-type (mean +/- S.E.M; wild-type n=3, IUE n=3). (G-H) Quantification of P2ry12+ particles (G, $p = 0.0004$) and P2ry12 particle area (H, $p = 0.0042$) within the cortex in IUE brains as compared to wild-type (mean +/- S.E.M; wild-type n=3, IUE n=3).

Rosin et al, Figure S4

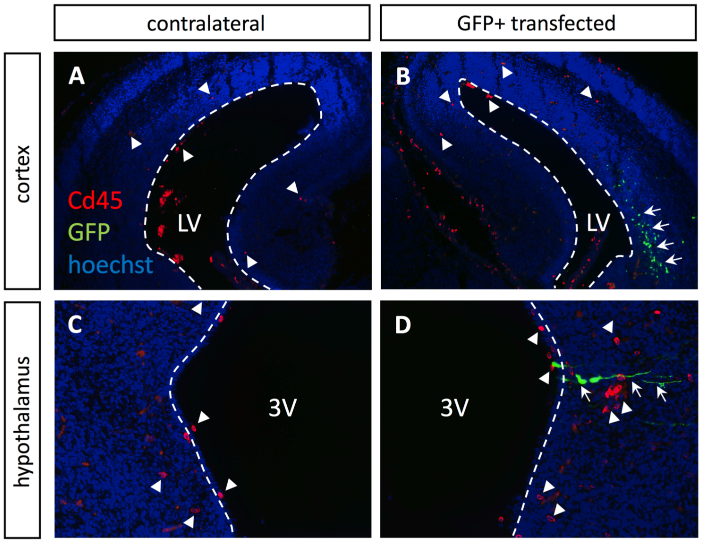

Supplementary Figure 4. *In utero* electroporation alters microglia morphology and expression signatures in both the transfected and contralateral side of the brain. E15.5 GFP and Cd45 expression in the contralateral (A, C) and GFP+ transfected (B, D) cortex (A, B) and hypothalamus (C, D) of *pCIG2* IUE (E14.5) embryos shows an increase in the number of Cd45+ cells. LV, lateral ventricles; 3V, third ventricle. Dashed-lines outline the ventricles. White arrows mark GFP+ cells (B, D), while white arrowheads mark Cd45+ cells (A-D).

Rosin et al, Figure S5

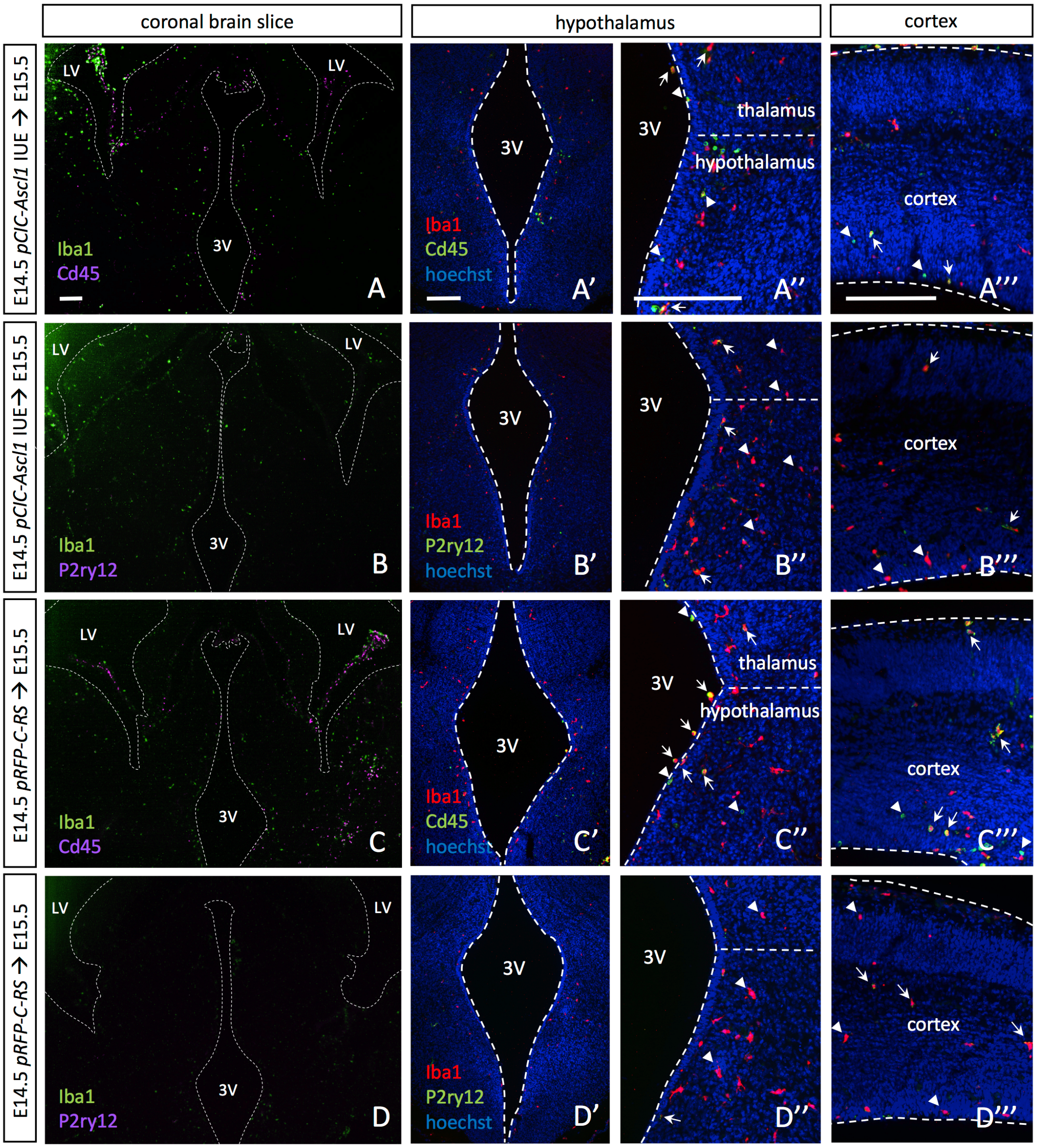

Supplementary Figure 5. *In utero* electroporation with *pCIC-Ascl1* or *pRFP-C-RS* also alters microglia expression signatures throughout the embryonic brain. (A) E15.5 *pCIC-Ascl1* IUE (E14.5) Iba1 and Cd45 expression in a coronal brain slice. Higher magnification images of E15.5 *pCIC-Ascl1* IUE (E14.5) Iba1 and Cd45 expression in the embryonic hypothalamus (A’, A’’) and cortex (A’’’). (B) E15.5 *pCIC-Ascl1* IUE (E14.5) Iba1 and P2ry12 expression in a coronal brain slice. Higher magnification images of E15.5 *pCIC-Ascl1* IUE (E14.5) Iba1 and P2ry12 expression in the embryonic hypothalamus (B’, B’’) and cortex (B’’’). (C) E15.5 *pRFP-C-RS* IUE (E14.5) Iba1 and Cd45 expression in a coronal brain slice. Higher magnification images of E15.5 *pRFP-C-RS* IUE (E14.5) Iba1 and Cd45 expression in the

embryonic hypothalamus (C', C'') and cortex (C'''). (D) E15.5 *pRFP-C-RS* IUE (E14.5) Iba1 and P2ry12 expression in a coronal brain slice. Higher magnification images of E15.5 *pRFP-C-RS* IUE (E14.5) Iba1 and P2ry12 expression in the embryonic hypothalamus (D', D'') and cortex (D'''). LV, lateral ventricle; 3V, third ventricle. Dashed-lines outline the ventricles, and mark the division between the thalamus and hypothalamus. White arrows mark Iba1+/Cd45^{high} double-positive (A''-A''', C''-C'''), or Iba1+/P2ry12+ double-positive (B''-B''', D''-D''') cells, while white arrowheads mark Cd45^{high} single-positive (A''-A''', C''-C'''), or Iba1+ single-positive (B''-B''', D''-D''') cells. Scale bar represents 250µm (A-D''').

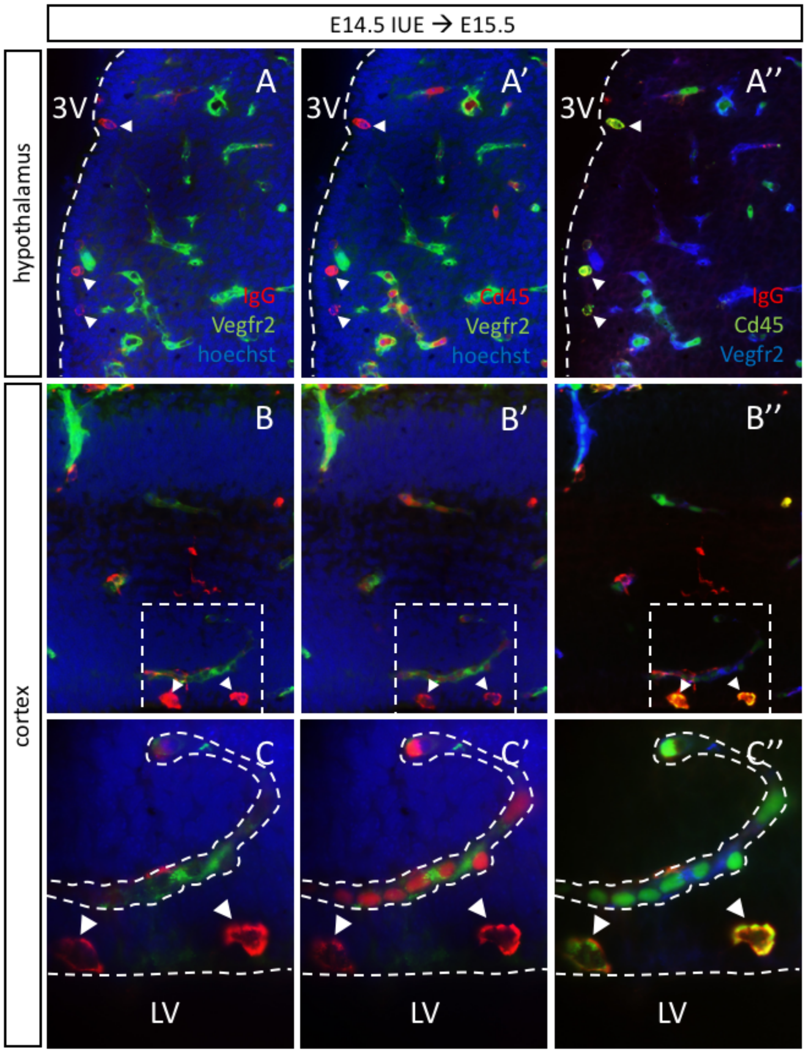

Supplementary Figure 6. IgG+ Cd45^{high} cells can be found outside of blood vessels and within the brain parenchyma. Expression of IgG and Vegfr2 (A, B, C), Cd45 and Vegfr2 (A', B', C'), or IgG, Cd45 and Vegfr2 (A'', B'', C'') in E15.5 *pCIG2-EGFP* IUE (E14.5) brains demonstrate that IgG+ and/or Cd45^{high} cells can be found outside of blood vessels (marked by Vegfr2) and lining the ventricles in the brain parenchyma of both the hypothalamus (A-A'') and cortex (B-C''). LV, lateral ventricle; 3V, third ventricle. Dashed-lines outline the ventricles, or mark blood vessels. White arrowheads mark monocyte-derived macrophages located outside of blood vessels.

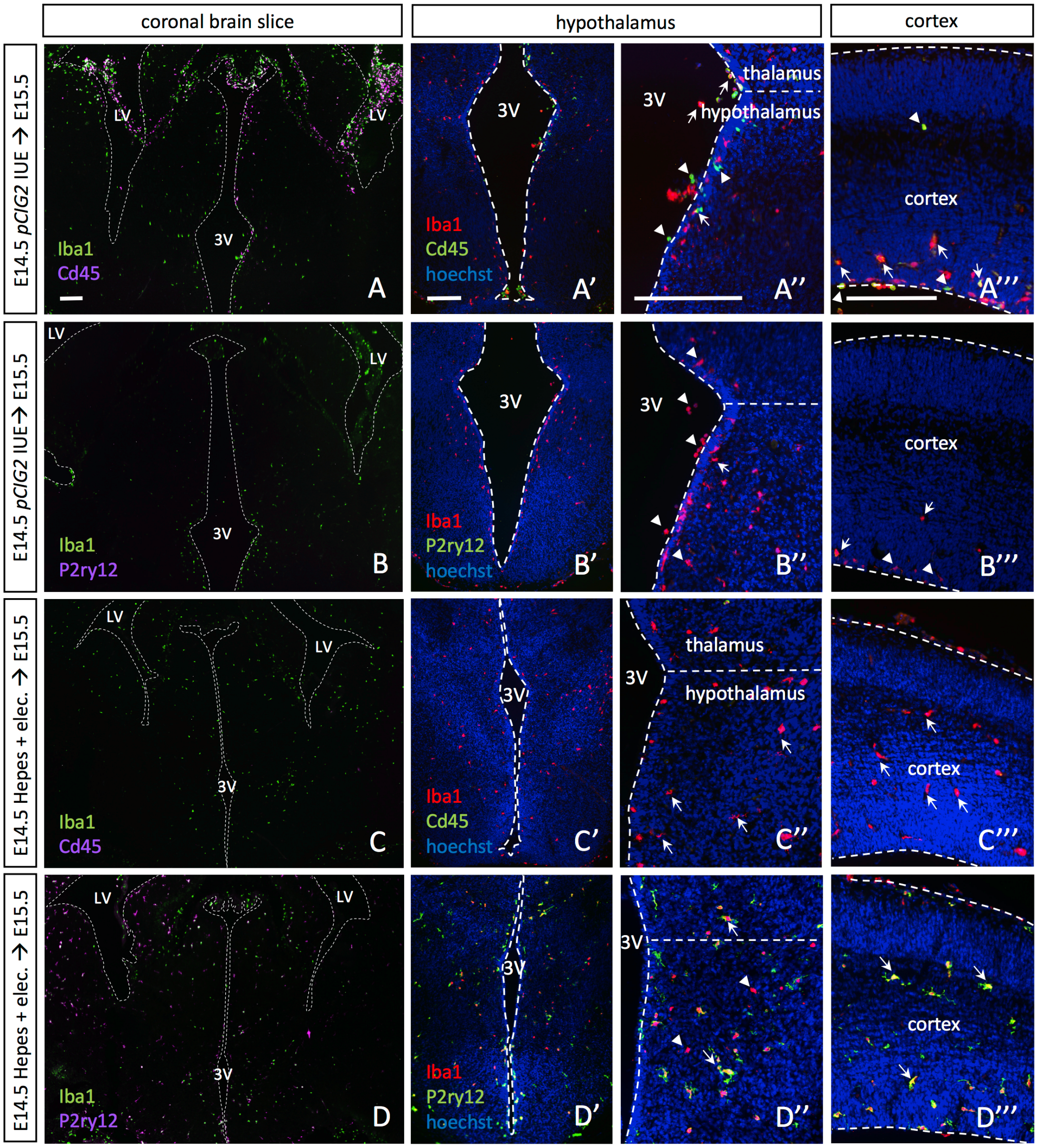

Supplementary Figure 7. ***In utero*** **electroporation using milder parameters still alters microglia expression signatures, yet the replacement of DNA with negatively charged Hepes does not.** (A-B''') E15.5 *pCIG2* IUE (E14.5; parameters: 500ng/µL, 30V, 50ms, 4 times, 1000 ms interval). (A) E15.5 *pCIG2* IUE (E14.5) Iba1 and Cd45 expression in a coronal brain slice. Higher magnification images of E15.5 *pCIG2* IUE (E14.5) Iba1 and Cd45 expression in the embryonic hypothalamus (A', A'') and cortex (A'''). (B) E15.5 *pCIG2* IUE (E14.5) Iba1 and P2ry12 expression in a coronal brain slice. Higher magnification images of E15.5 *pCIG2* IUE (E14.5) Iba1 and P2ry12 expression in the embryonic hypothalamus (B', B'') and cortex (B'''). (C) E15.5 Iba1 and Cd45 expression in a coronal brain slice from an embryo injected with 1M Hepes prior to electroporation. Higher magnification images of E15.5 Iba1 and Cd45 expression

in the embryonic hypothalamus (C', C'') and cortex (C'''). (D) E15.5 Iba1 and P2ry12 expression in a coronal brain slice from an embryo injected with 1M Hepes prior to electroporation. Higher magnification images of E15.5 Iba1 and P2ry12 expression in the embryonic hypothalamus (D', D'') and cortex (D'''). LV, lateral ventricle; 3V, third ventricle. Dashed-lines outline the ventricles, and mark the division between the thalamus and hypothalamus. White arrows mark Iba1+/Cd45^{high} double-positive (A''-A'''), Iba1+ single-positive (C''-C'''), or Iba1+/P2ry12+ double-positive (B''-B''', D''-D''') cells, while white arrowheads mark Cd45^{high} single-positive (A''-A'''), or Iba1+ single-positive (B''-B''', D''-D''') cells. Scale bar represents 250μm (A-D''').

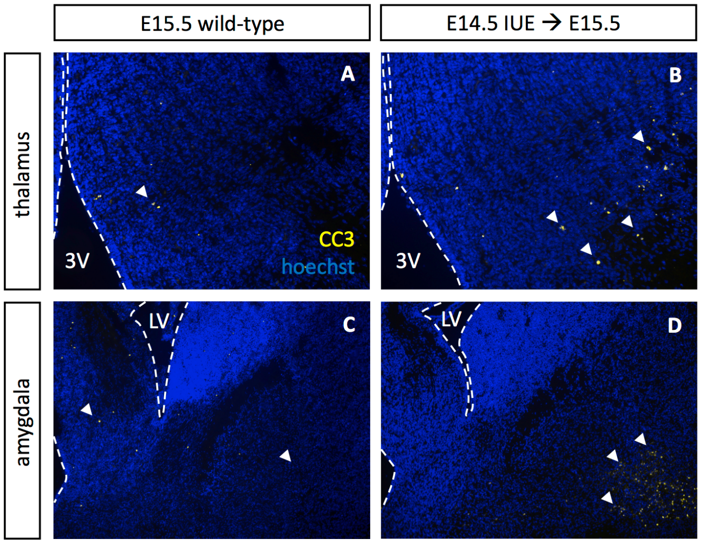

Supplementary Figure 8. *In utero* electroporation can induce cell death in the developing thalamus and amygdala. Expression of active cleaved Caspase 3 (CC3) in E15.5 wild-type (A, C) and *pCIG2* IUE (E14.5) brains (B, D) highlights the increase in cell death in the thalamus (B, n = 2 / n = 3 analyzed) and amygdala (D, n = 1 / n = 3 analyzed) of IUE brains. LV, lateral ventricle; 3V, third ventricle. Dashed-lines outline the ventricles. While arrowheads mark CC3+ cells.

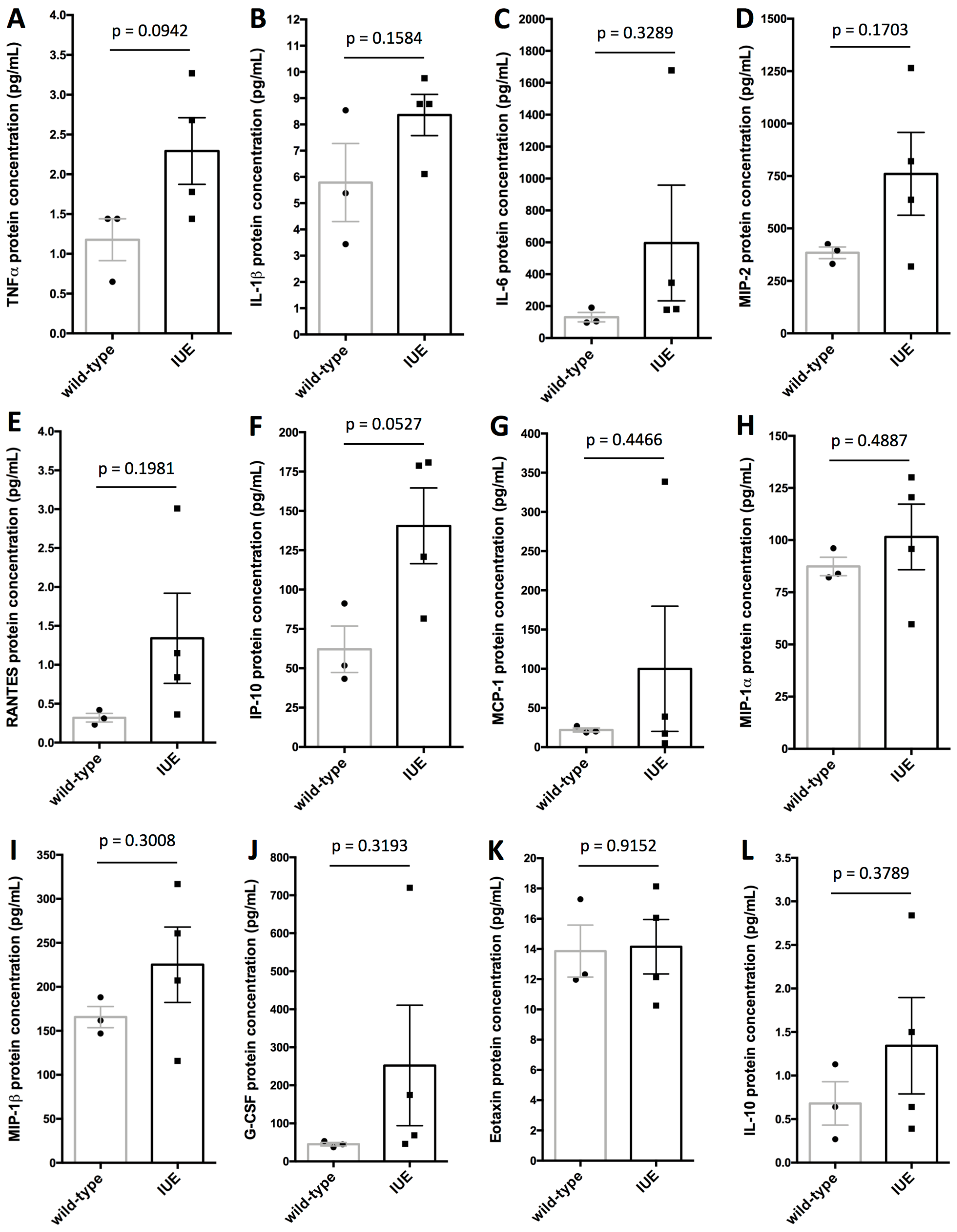

Supplementary Figure 9. Cytokine and chemokine levels in the E17.5 brain following *in utero* electroporation at E14.5. Cytokine and chemokine levels of (A) Tumor necrosis factor alpha (TNFα; p = 0.0942), (B) Interleukin 1 beta (IL-1β; p = 0.1584), (C) Interleukin 6 (IL-6; p = 0.3289), (D) Macrophage inflammatory protein 2 (MIP-2; p = 0.1703), (E) Regulated upon activation normal T cell expressed and secreted (RANTES, also known as CCL5; p = 0.1981),

(F) Interferon gamma-induced protein 10 (IP-10, also known as CXCL10; $p = 0.0527$), (G) Monocyte chemotactic protein 1 (MCP-1, also known as CCL2; $p = 0.4466$), (H) Macrophage inflammatory protein 1 alpha (MIP-1α, also known as CCL3; $p = 0.4487$), (I) Macrophage inflammatory protein 1 beta (MIP-1β, also known as CCL4; $p = 0.3008$), (J) Ganulocyte colony-stimulating factor (G-CSF; $p = 0.3193$), (K) Eotaxin ($p = 0.9152$), and (L) Interleukin 10 (IL-10; $p = 0.3789$) in E17.5 IUE (E14.5) brains as compared to wild-type (protein levels pg/mL; mean +/- S.E.M.; wild-type n=3, IUE n=4).
